# Modeling Tay-Sachs Disease in Astrocyte-like Cells Reveals Significant Changes in the Transcriptomic Profile

**DOI:** 10.3390/ijms27146503

**Published:** 2026-07-22

**Authors:** Diego A. Suárez-García, Angela J. Espejo-Mojica, Carlos J. Alméciga-Díaz

**Affiliations:** Institute for the Study of Inborn Errors of Metabolism, Faculty of Science, Pontificia Universidad Javeriana, Bogotá D.C. 110231, Colombia; suarezd.i@javeriana.edu.co (D.A.S.-G.); aespejo@javeriana.edu.co (A.J.E.-M.)

**Keywords:** Tay-Sachs, astrocyte, lysosomal storage disease, CRISPR

## Abstract

Tay-Sachs disease is a rare genetic disorder characterized by the accumulation of GM2 ganglioside in neuronal lysosomes due to deficient β-hexosaminidase A (HexA) activity. Progressive GM2 storage leads to severe neurodegeneration, including developmental delay, motor weakness, seizures, ataxia, and early death, typically by five years of age. Previous studies have elucidated several neuronal mechanisms, including apoptosis, endoplasmic reticulum stress, neuroinflammation, and demyelination, these investigations have focused almost exclusively on neurons. However, other components of the central nervous system, particularly astroglia, may play a critical role in disease pathophysiology as suggested by studies in related lysosomal storage disorders. To address this gap, we generated an astrocyte-like model deficient in HexA by targeted knockdown of the *HEXA* gene in U87MG astrocytoma cells. The resulting cell line recapitulates key pathological features, including lysosomal accumulation, increased neutral lipid content, reduced mitochondrial mass, and elevated reactive oxygen species production. Transcriptomic analysis revealed significant alterations in pathways associated with neuronal degeneration, synaptic organization, mitochondrial dysfunction, and ganglioside metabolism. In summary, this model reproduces some classical cellular alterations reported in Tay-Sachs disease and could potentially provide novel insight into astrocyte involvement in its pathophysiology. These findings support the relevance of non-neuronal cells in disease pathophysiology and establish this system as a valuable platform for screening potential novel mechanisms and therapeutic approaches. Furthermore, this approach highlights the importance of integrating cell type specific models to better understand disease heterogeneity and providing insights into the progressive neurodegeneration of Tay-Sachs disease, positioning this model as a valuable tool for studying its underlying pathophysiology.

## 1. Introduction

Tay-Sachs disease (TSD) is a rare hereditary neurodegenerative disorder classified within the group of gangliosidoses. It is characterized by the pathological accumulation of gangliosides in neuronal tissue, a hallmark feature of this class of lysosomal storage diseases [1]. Mutations on both alleles of the *HEXA* gene (chromosome 15q23) are responsible for β-hexosaminidase A (HexA) deficiency [1]. This enzyme catalyzes the conversion of GM2 into GM3 ganglioside by cleaving the terminal N-acetylgalactosamine (GalNAc) residue from GM2, thereby facilitating the stepwise degradation of complex glycosphingolipids within neuronal tissue [2]. Without proper enzymatic degradation, GM2 accumulation occurs, and symptoms begin to manifest most often in the first year of life.

The global incidence of TSD is estimated at approximately 1 in 320,000 live births, with a reported incidence in the United States of 1 in 100,000 live births. A markedly higher frequency is observed among individuals of Ashkenazi Jewish descent, where the incidence reaches up to 1 in 3500 live births. Elevated prevalence has also been documented in specific founder populations, including the Cajun community in Louisiana, the Amish population in Pennsylvania, and non-Jewish French-Canadians [2,3,4,5].

Three distinct clinical phenotypes of TSD have been described: infantile, juvenile, and adult-onset. Each form is characterized by differences in age of onset, clinical progression, and severity, reflecting the heterogeneity of GM2 gangliosidosis manifestations [6]. The infantile form is the most frequent and severe presentation with premature death in the first 5 years of age and clinical manifestations such as neurodevelopmental delay, exaggerated startle response, ataxia, loss of motor function, seizures, difficult to swallow, macrocephaly, and blindness [7]. A cherry-red macular spot is a classic finding in TSD patients [7]. The juvenile and adult-onset forms of TSD exhibit a delayed clinical onset and are generally associated with a less aggressive symptomatology compared to the infantile phenotype [6,8]. While the juvenile phenotype typically progresses to a vegetative state, characterized by decerebrate posturing, with death commonly occurring between 10 and 15 years of age; the adult-onset phenotype is frequently associated with psychiatric manifestations, including depression, schizophrenia, and dementia, which usually emerge between the ages of 20 and 30 years [9].

Several studies have focused on how GM2 ganglioside built up affects neurons [1]. Most important findings suggest that GM2 can escape the lysosome and interact with different cell components triggering processes such as apoptosis, endoplasmic reticulum stress, calcium altered homeostasis, demyelination and neuroinflammation [1]. Microgliosis and immune cell infiltration have also been documented in TSD murine models, suggesting that neuroinflammation is, at least in part, a consequence of microglial activation in response to neuronal death [10].

Several TSD animal models have been developed [11]. Key findings in the TSD murine models include reduced HexA activity, increased proinflammatory cytokines in brains from affected mice, microglial activation, decreased levels of glycerophospholipids (phosphatidylethanolamine, phosphatidylcholine, phosphatidylinositol, and phosphatidylserine), skeletal abnormalities, progressive neurodegeneration, redox homeostasis imbalance, progressive loss of reflexes, ataxia and premature death [12,13,14,15,16]. A Jacob sheep TSD model resembles juvenile and adult TSD showing GM2, ceramide, and GM3 ganglioside increase in brain [17]. Additionally, this model presents symptoms related to neuronal deterioration such as strabismus, decreased muscle tone, and proprioceptive deficit [17,18]. In 2013, Rickmeyer et al. reported a spontaneous TSD rabbit model exhibiting abnormal gait, decreased sway, clasping of the limbs and increased cervical lordosis [19].

Cellular models for the study of TSD include skin fibroblast and induced pluripotent stem cells (iPSCs) [11]. Skin fibroblast from TSD patients exhibits lamellar structures embedded within membranous compartments of lysosomal origin, autophagic flux inhibition, lysosome membrane permeabilization and increased oxidative stress [20,21,22,23]. iPSCs derived from skin fibroblast show no alteration of autophagic flux and increase sensitivity to induced oxidative stress [24]. Similarly, neuronal stem cells differentiated from TSD iPSCs did not show altered autophagic state but an altered synaptic exocytosis revealing a novel mechanism explaining TSD physiopathology [25].

Despite substantial evidence demonstrating that HexA deficiency disrupts neuronal homeostasis and implicates microglial activation in this process, the contribution of astroglia to disease pathogenesis remains insufficiently explored. A study conducted in a murine model of Sandhoff disease (*HEXB*^-/-^) demonstrated a marked increase in reactive astrocytes (astrogliosis), with astrocyte activation showing to be dependent on FcRγ receptor signaling. Moreover, modulation of immune responses attenuated astrocytic activation and resulted in improved motor performance in treated animals [26]. Altogether, this suggests that astrocyte could contribute to neurological degeneration in classical TSD patients and it could be a potential therapeutic target for future treatment strategies. In this study, we developed and characterized a HexA deficient cellular model using an astrocyte-like cell line (U87MG) to explore the impact of HexA deficiency on the molecular and cellular mechanisms.

## 2. Results

### 2.1. HEXA KO on U87MG Cells by CRISPR-Cas9 Editing

U87MG is one of the most extensively utilized glioblastoma cell lines [27] and has been employed in studies addressing different neurodegenerative disorders [28,29,30,31]. In this study, we generated a *HEXA* knock-out model in U87MG cells using the CRISPR-Cas9 system.

**Figure 1:**
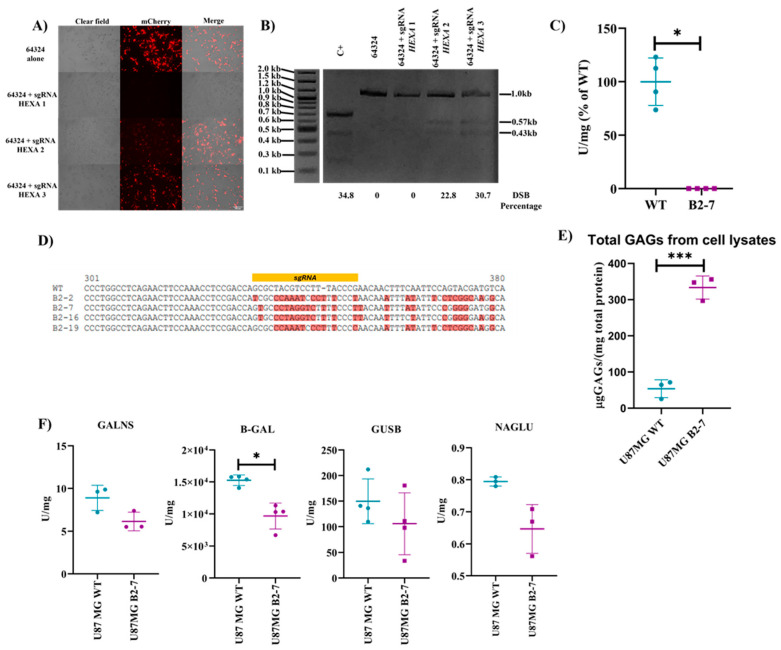
Transfection of U87MG cells with a CRISPR-Cas9 plasmid to generate *HEXA* KO clones. (**A**) HEK 293 FT cells were transfected with CRISPR-Cas9 plasmid holding sgRNA sequences targeting *HEXA* gene first exon. mCherry reporter gene induced red fluorescence on transfected cells. The images were acquired through epifluorescence microscopy. (**B**) Genomic DNA from transfected cells was extracted and a region flanking the CRISPR-Cas9 predicted cutting site was PCR amplified. PCR fragments were subsequently submitted to T7 assay to estimate double strand break (DSB) percentage. (**C**) Enzymatic HexA activity was measured for verification of HexA deficiency in B2-7 clone (purple) compared to wild-type (WT, cyan) cells. Results of HexA enzymatic activity are expressed as mean ± SD of four independent experiments. Kolmogorov-Smirnov test. * *p* < 0.05. (**D**) Genomic DNA from clones with lower or no enzymatic activity was isolated and sequenced by the sanger method. The yellow bar indicates sgRNA sequence. Red boxes indicate mismatched nucleotides compared to the wild type sequence. (**E**) Total GAGs in cell lysates collected from U87MG WT (cyan) and B2-7 (purple) cell cultures. Data expressed as mean ± SD of three independent experiments. Unpaired *t* test *** *p* < 0.0005. (**F**) Enzymatic activity of GALNS, B-GAL, GUSB and NAGLU from cell lysates of U87MG WT (cyan) and U87MG B2-7 (purple) cell cultures. Enzymatic activity differences were estimated from three or four independent replicates and expressed as mean ± SD. * *p* < 0.05 Mann-Whitney test.

First, we designed and selected sgRNA sequences based on their high specificity and predicted low off-target effects [32] (Supplementary Table S1). After selecting and cloning three sgRNAs into a CRISPR-Cas9 plasmid (Addgene 64324, Supplementary Figure S2C), we evaluated on-target activity of the designed sgRNAs. HEK 293FT cells were transfected with CRISPR-Cas9/sgRNA plasmids, and T7 endonuclease assay was performed 48 h post-transfection (Figure 1A). Red fluorescence in the cell cytoplasm indicates successful transfection and expression of the Cas9/sgRNA complex, suggesting that the cell is likely to undergo a double-strand break (DSB) at the targeted genomic locus. The plasmid carrying the sgRNA1 sequence did not exhibit a noticeable increase in fluorescence, suggesting inefficient transfection. Two of the three plasmid constructs showed cleavage activity, with 64324 + sgRNA *HEXA* 3 exhibiting the highest on-target activity, which was selected for subsequent experiments (Figure 1B).

Transfection of the plasmid 64324 + sgRNAs *HEXA 3* on U87MG cells was confirmed by mCherry reporter expression and yielded ~3% efficiency as determined by flow cytometry (Supplementary Figure S3). mCherry-positive cells were sorted and clonally expanded. Among the isolated clones, four displayed markedly reduced HexA activity (<5% of wild type) (Figure 1C and Figure S4A). Sanger sequencing revealed frameshift mutations in exon 1 of *HEXA* in the four clones with the lowest activity (Figure 1D and Figure S5).

Since β-hexosaminidases play a secondary role in the enzymatic degradation of glycosaminoglycans (GAGs) [33], we measured the total GAGs content in the culture medium and cell lysates from four U87MG clones with reduced HexA activity. Among these different HEXA knockout clones, only two exhibited significant differences compared with the wild-type cells (Figure 1E and Figure S4B).

In addition to the loss of HexA activity, U87MG B2-7 cells exhibited reduced activity of other lysosomal hydrolases (GALNS, B-Gal, GUSB, and NAGLU, Figure 1F), suggesting that *HEXA* deficiency is associated with a broader impairment of lysosomal function rather than an isolated enzymatic defect. This observation is consistent with the transcriptomic analysis (see Section 2.5), which revealed downregulation of multiple genes associated with lysosomal biology, including lysosomal hydrolases, membrane transporters, and regulators of lysosome biogenesis. Together, these findings demonstrate the successful generation of a *HEXA* knockout in U87MG cells, which recapitulates the enzymatic deficiency underlying TSD. In addition, these results indicate that HexA deficiency is accompanied by coordinated alterations affecting lysosomal homeostasis.

### 2.2. Characterization of HEXA KO Cells

**Figure 2:**
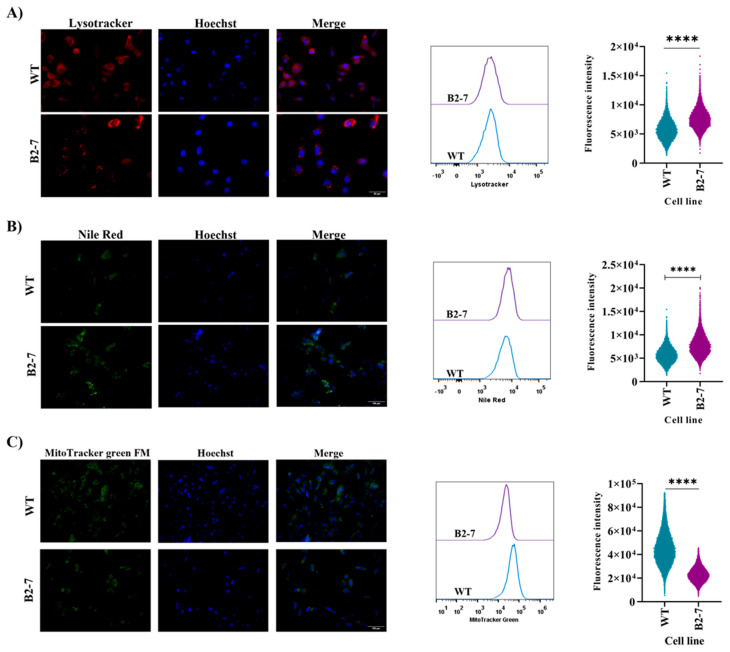
Characterization of the U87MG *HEXA* KO model. (**A**) Lysotracker Deep Red staining for Lysosomal mass visualization on epifluorescence microscopy (Left 40×) and quantification by flow cytometry (Right). (**B**) Nile Red staining for Neutral lipids labeling on epifluorescence microscopy (Left 20×) and quantification by flow cytometry (Right). (**C**) Cells labeled with MitoTracker green FM for mitochondria staining on epifluorescence microscopy (Left 20×) and quantification by flow cytometry (Right). Color code: wild-type (WT) cells, cyan; and B2-7 clone, purple. Data are presented as mean ± SEM. Statistical significance was assessed using Mann-Whitney test (**** *p* < 0.0001).

The U87MG B2-7 clone was selected for further evaluation since it showed the most consistent enzymatic reduction over different time measurements (Supplementary Figure S6). Since lysosomal accumulation is a classical hallmark of lysosomal storage disorders (LSDs) such as TSD, it is expected that *HEXA* KO cells exhibit this pathological trait. U87MG B2-7 cells were labeled with lysotracker deep red for visualization and quantification of lysosomal vesicles (Figure 2A). Epifluorescence microscopy revealed a distinct fluorescence pattern: while WT cells exhibited dispersed signal, B2-7 cells showed high-intensity fluorescence concentrated throughout the cytoplasm (Figure 2A, Left). Flow cytometry confirmed a significant increase in mean fluorescence intensity in B2-7 cells compared to WT cells (Figure 2A, Right). A similar pattern was observed when cells were labeled with Nile Red, which selectively stains molecules of lipidic nature such as gangliosides (Figure 2B). Consistent with this finding, Espejo-Mojica et al. reported elevated Nile red staining in TSD fibroblasts compared to a WT fibroblast [34].

We used TSD fibroblasts (GM00515B) and healthy fibroblast (GM23963C) as a reference model, given their widespread use in studies investigating cellular alterations associated with HexA deficiency and the evaluation of therapeutic strategies [20,21,35]. Initial assessment of HexA enzymatic activity confirmed the characteristic enzyme deficiency in the TSD fibroblasts (Supplementary Figure S7A). The increase in lysosomal mass observed in U87MG B2-7 cells was consistent with that detected in fibroblasts derived from a TSD patient compared to healthy fibroblasts, thereby reinforcing the validity of U87MG B2-7 cellular model in recapitulating key pathological features of HexA deficiency (Supplementary Figure S7B).

There is also evidence that mitochondrial dysfunction is linked to neurological degeneration in LSDs [36]. In this sense, MitoTracker Green FM staining revealed a significant decrease in mitochondrial mass in U87MG B2-7 cells relative to WT cells (Figure 2C). This change contrasted with TSD fibroblasts, where an increased mitochondrial mass was observed (Supplementary Figure S7C). These results suggest that *HEXA* KO increases lysosomal mass due to lipids accumulation, consistent with TSD patient derived fibroblasts. However, the distinct mitochondrial phenotype in U87MG B2-7 cells indicates that *HEXA* KO alters mitochondrial homeostasis differently compared to TSD fibroblasts.

### 2.3. Mitochondrial and General Oxidative Stress State Change in U87MG HEXA KO Cells

**Figure 3:**
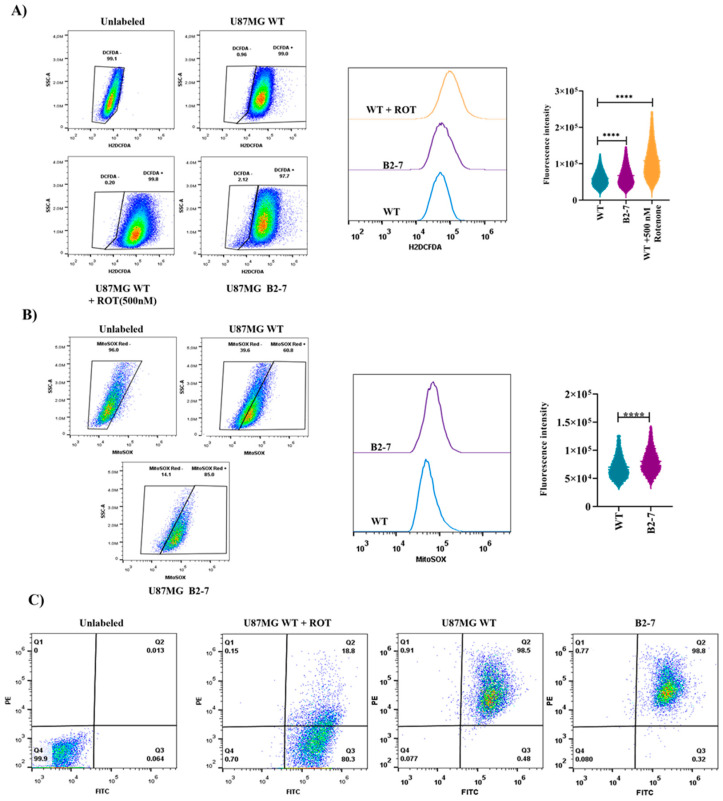
Oxidative stress evaluation on U87MG *HEXA* KO model. (**A**) H_2_DCFDA staining for ROS measurement on U87MG *HEXA* KO cells by flow cytometry. Dot plots show increase on fluorescence intensity (Left). Histograms of positive stained cells (middle). Graph showing fluorescence distribution of each cell population (Right). Positive control with rotenone, a specific inhibitor of the respiratory chain’s first complex, indicates proper labeling of ROS since inhibition of complex I should result on increased fluorescence signal. (**B**) Measurement of MitoSOX fluorescence intensity on U87MG *HEXA* KO cells by flow cytometry. Dot plots of labeled cells with MitoSOX reagent evaluated by flow cytometry (Left). Histograms of positive stained cells (middle). Graph showing fluorescence distribution of each cell population (Right) (**C**) Representative quadrant plots of non-labeled cells, cell treated with Rotenone (ROT), WT cells, and *HEXA KO* cells (B2-7). X axis represents FITC fluorescence and Y axis represent PE fluorescence. Color code: wild-type (WT) cells, cyan; B2-7 clone, purple; and WT cells treated with ROT), orange. Data is presented as mean ± SEM. Statistical significance was assessed using Mann-Whitney test (**** *p* < 0.0001) or Kruskal-Wallis test (**** *p* < 0.0001) followed by Dunn’s multiple comparison test.

Increased ROS is a common hallmark found in many LSDs due to accumulated macromolecules in lysosomes [37]. Other changes linked to lysosomal dysfunction include a decrease in mitochondrial membrane potential [38]. Given the alterations observed in U87MG B2-7 mitochondrial mass, we further explored the total ROS levels in these cells (Figure 3A). In this sense, the U87MG B2-7 clone exhibited an approximately 13% increase in fluorescence intensity compared with the WT signal (Figure 3A). Consistently, mitochondrial-associated superoxide production—previously reported in other LSDs [21,38] was in line with the observed rise in mitochondrial-associated ROS levels in U87MG B2-7 cells (15% higher than in WT cells) (Figure 3B). As mentioned before, mitochondrial membrane potential is associated with ROS production in LSDs [38]; however, results showed minor differences in membrane potential between the U87MG B2-7 clone and WT cells (Figure 3C). A similar result on general ROS, mitochondrial ROS and mitochondrial membrane potential was observed in TSD skin-fibroblasts (Supplementary Figure S8).

In summary, these results indicate that *HEXA* KO does not affect the mitochondrial membrane of either U87MG B2-7 or TSD skin fibroblasts, and that the increase in mitochondrial-associated oxidative stress seems to be associated with a mechanism that does not involve impaired membrane potential.

### 2.4. Endoplasmic Reticulum Evaluation Reveals Increase ER Mass

**Figure 4:**
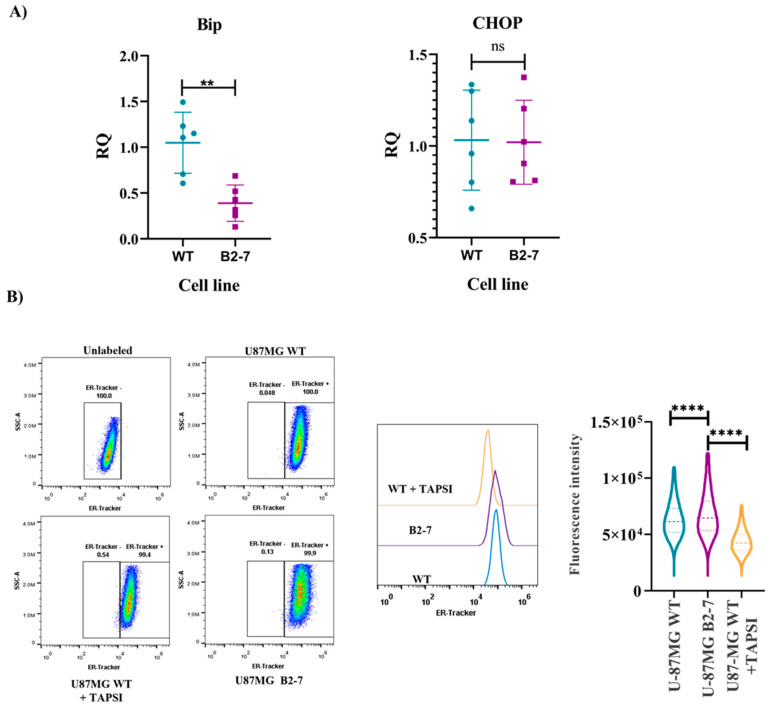
Relative quantification (RQ) of genes related to ER-associated stress. RQ represents fold change compared to the wild type control. Differences were estimated from six replicates. Data are presented as mean ± SEM. Statistical significance was assessed using the Mann-Whitney test. Color code: wild-type (WT) cells, cyan; and B2-7 clone, purple (**A**). ER-Tracker evaluation by flow cytometry on U87MG WT and U87MG B2-7 cells. Color code: wild-type (WT) cells, cyan; B2-7 clone, purple; and WT cells treated with TAPSI, orange (**B**). Differences were estimated from three independent replicates. Data are presented as mean ± SEM. Statistical significance was assessed using the Kruskal–Wallis test followed by Dunn’s multiple comparison test (** *p* < 0.01, **** *p* < 0.0001). ns = not significant.

GM2 accumulation can disrupt lysosomal homeostasis and induce its release into the cellular cytoplasm affecting other organelles such as endoplasmic reticulum (ER) [1]. Prolonged interaction of GM2 with ER can induce ER-stress by activation of proapoptotic proteins such as CHOP [1]. We investigated changes in the ER-stress state, specifically, we explored changes in the unfolded protein response (UPR) due to deficiency of HexA. The results showed that there is no change in the gene expression of CHOP and Bip proteins which are important mediators of the UPR (Figure 4A). Similarly, other genes such as *ATF6*, *ATF4*, *ERN1*, and *XBP1* which are also involved in ER-stress were not overexpressed either (Supplementary Figure S9). In contrast, an increase in U87MG B2-7 clone ER-mass was observed compared to WT cells (Figure 4B). Our findings did not reveal overexpression of ER stress–related genes; however, it was observed an increase in ER mass; which may indicate ER membrane sequestration of accumulated lipids as a consequence of impaired HexA function.

### 2.5. Differential Expression Analysis Reveals Significant Divergence in Gene Expression Profile of HEXA Knocked out Cells

**Figure 5:**
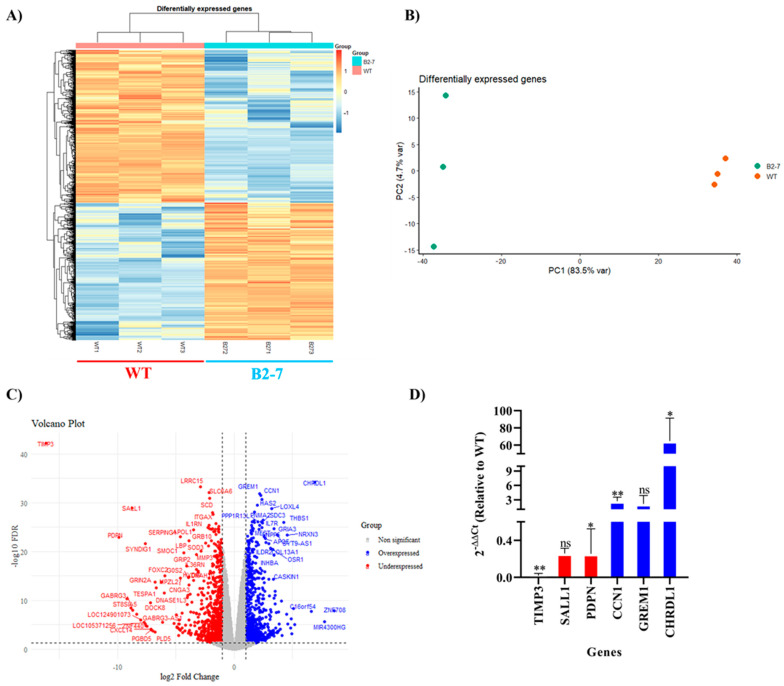
Transcriptomic analysis from RNA samples extracted from non-mutated cells (WT) and HEXA Knocked-out cells (B2-7). (**A**) Heatmap constructed by hierarchical Clustering of differentially expressed genes of B2-7 cells vs. control cells using the Euclidian distance method. Orange bars represent overexpressed genes while blue bars are downregulated genes. (**B**) Principal component analysis (PCA) of differentially expressed genes. Each point represents a replicate sample from B2-7 cells (green) or WT cells (orange) along PC1 and PC2. (**C**) Volcano plot of differentially expressed genes. Each point represents a gene. Genes with the highest or lowest expression are labeled. Blue dots represent overexpressed genes (log Fold Change > 1) and red dots represent downregulated genes (log Fold Change < −1). Highlighted genes are those with a False Discovery Rate (FDR) < 0.05. (**D**) RT-qPCR of different genes for transcriptome validation. Genes were normalized to a *GAPDH* as housekeeping gene. Relative expression expressed as fold change (2^−∆∆Ct^ relative to WT) by de ∆∆Ct method. Color code: underexpressed genes, red; overexpressed genes, blue. Statistical significance was assessed using the Mann-Whitney test (* *p* < 0.05, ** *p* < 0.01). ns = not significant.

Previous reports have explored gene expression profile changes due to non-functional HexA to uncover cellular mechanisms that could explain the progressive neurodegeneration in TSD patients [39,40,41]. In this sense, we evaluated the transcriptomic changes on the U87MG B2-7 compared to WT cells, as well as with results from other studies [39,40,41]. From total RNA samples, we obtained total raw counts from 62,754 transcript counts that were correctly aligned to an annotated genome region and presented high quality scores (Supplementary Figure S10A). Normalization of these annotated counts resulted in similarly distributed counts among the samples (Supplementary Figure S10B). Cluster analysis grouped the six samples in two groups in which WT samples are separated from U87MG B2-7 clone suggesting a divergent gene expression profile between these populations (Figure 5A). Principal component analysis clearly separated U87MG B2-7 from WT samples, with two principal components representing 88.2% of the total variance (Figure 5B). This means that expression of genes in U87MG B2-7 clone significantly differs from that of WT cells. From differential expression analysis, it was observed that 4124 and 4345 genes were significantly upregulated and downregulated, respectively, in the U87MG B2-7 clone compared to WT cells (Figure 5C) [42,43,44].

To further validate the RNA-seq results, we performed RT-qPCR analysis of six representative differentially expressed genes (*TIMP3*, *SALL1*, *PDPN*, *CCN1*, *GREM1*, and *CHRDL1*). These genes were selected because they are representative of several of the biological processes identified by transcriptomic analysis. RT-qPCR confirmed the direction of expression changes observed in the RNA-seq analysis for all six genes (Figure 5D), supporting the robustness of the transcriptomic data set.

### 2.6. Functional Enrichment Analysis Uncovers Processes Related to Neuronal Development

**Figure 6:**
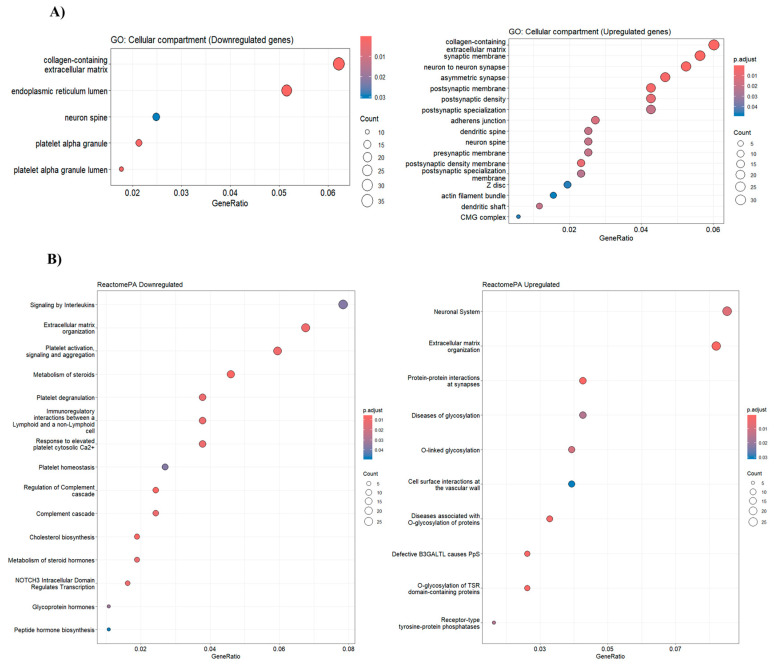
Gene ontology (GO) and Reactome analysis. (**A**) GO focused on cellular component (CC) terms shows genes with most variable expressions on *HEXA* Knocked-out cell (U87MG) compared to the control (WT). (**B**) Dot plot enrichment analysis based on Reactome pathways. Dot plots of downregulated genes are shown on the left and Dot plots of upregulated genes are on the right. Gene ratios on x axis are the number of genes on each CC term divided by the total number of genes evaluated. Dots size represents the number of genes involved with each BP term while color indicates *p*-values. *p*-values < 0.05 are considered statistically significant.

TSD is characterized by progressive deterioration of the CNS because of chronic inflammatory state leading to neuronal death [45]. Evaluation of functional enrichment analysis revealed several GO terms for the upregulated genes associated with biological processes related to neuronal development and differentiation such as axogenesis, regulation of neuron projection development, axon guidance, neuron projection guidance, synapse assembly, and regulation of synapse organization (Supplementary Figure S11, Right). Downregulated genes were mainly associated with processes not strictly related to neurodevelopment such as cell migration and metal ions transport (Supplementary Figure S11, Left). GO analysis focused on cellular compartment terms showed a similar output in which most upregulated genes were associated with GO terms related to neural function. For example, strong enrichment was observed for synapse membrane, neuron to neuron synapses, asymmetric synapse, and dendritic spine. Downregulated genes were found to be related to cellular compartments such as extracellular matrix, ER lumen and neuron spine (Figure 6A). Reactome pathways showed that the differentially expressed upregulated genes in the U87MG B2-7 clone are related to proper synapse assembly (Figure 6B), while downregulated genes are related to pathways such as extracellular matrix organization, interleukin signaling, cholesterol, and biosynthesis. Since the GM2 ganglioside is part of the lipid rafts on the cell membranes of neurons, the alteration of pathways related to the extracellular matrix homeostasis is consistent with previous reports [46]. Finally, functional enrichment analysis using KEGG pathway terms showed that genes related to PI3K-AKT and FoxO signaling are upregulated in U87MG B2-7 clone (Supplementary Figure S12). These signaling pathways are involved in processes such as neuronal stem cell proliferation, neuronal maturation, migration, and apoptosis [47].

### 2.7. Gene Expression Profiles Related to Key Biological Processes on HEXA Knocked-Out Cells Are Altered

**Figure 7:**
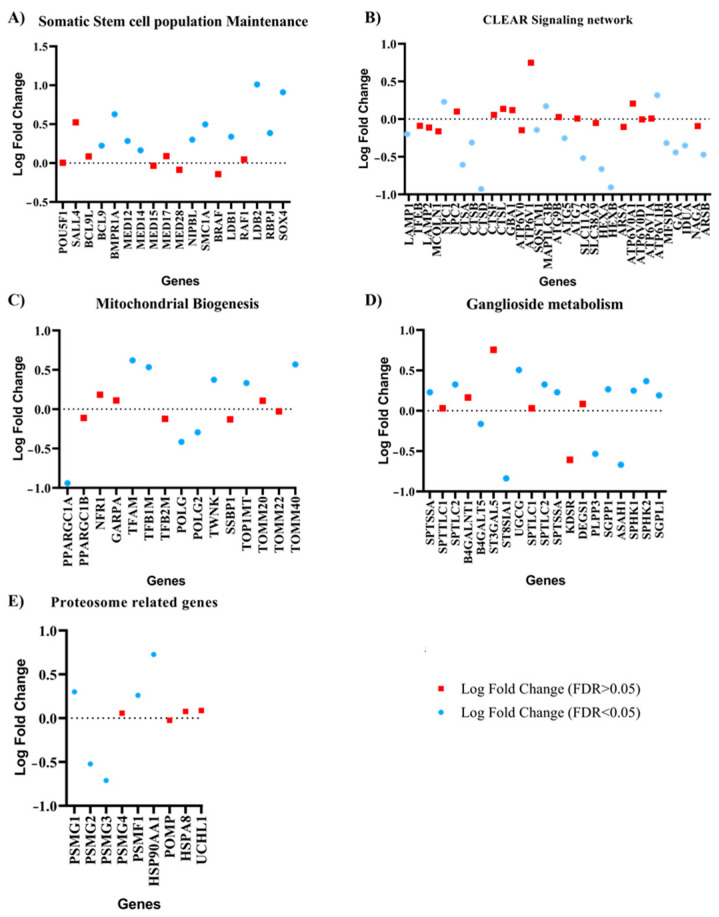
*HEXA* knock-out cells gene expression profiles of different gene sets involved in (**A**) Somatic Stem Cell Maintenance, (**B**) Lysosomal biogenesis, (**C**) Ganglioside metabolism, (**D**) Mitochondrial biogenesis and (**E**) Proteosome biogenesis. Gene expression is presented as Log_10_ Fold change (compared to control). Statistical significance in gene expression change is considered at FDR < 0.05.

Han et al., 2023 evaluated gene expression changes in human fetal brains from TSD patients and reported alterations on gene expression related to ganglioside metabolism, stem cell maintenance, and CLEAR signaling associated with lysosomal biogenesis [39]. To correlate these results with the functional enrichment analysis conducted in this study, we evaluated sets of genes reported to be altered in TSD by different studies [39,48]. Ten out of 18 genes involved in somatic stem cell maintenance showed strong upregulation in U87MG B2-7 clone compared to WT cells (Figure 7A), which correlates with the findings reported by Han et. al. [39].

The expression of most genes related to lysosomal biogenesis was downregulated (Figure 7B). Of the 33 evaluated genes, 13 were significantly downregulated in the U87MG B2-7 clone compared to WT cells, while only three were significantly upregulated. Interestingly, *TFEB*, which is the major regulator of lysosomal biogenesis, did not change significantly, while *LAMP1*, a glycoprotein commonly found in lysosomal membranes and maintains the structural integrity of lysosomes and endosomes, presents a lower gene expression in the U87MG B2-7 clone than in WT cells suggesting decreased lysosomal assembly as a countermeasure to disrupted lysosomal homeostasis. It has also been reported that impaired mitophagy due to altered lysosomal homeostasis could result in mitochondrial dysfunction in TSD [37]. Transcriptomic analysis showed considerable downregulation of *PPARGC1A*, the master regulator of mitochondrial biogenesis, in U87MG B2-7 [49] (Figure 7C), suggesting mitochondrial biogenesis inhibition as a compensatory mechanism for mitochondrial dysfunction.

Since HexA deficiency alters normal ganglioside catabolism, it is expected that several genes are also expected to be altered due to abnormal GM2 accumulation. Indeed, it has been reported that genes associated with ganglioside metabolism show differential gene expression in brain tissues from TSD patients [39]. In this sense, we observed that most genes involved in ganglioside biosynthesis were upregulated in U87MG B2-7, which includes *SPTSSA*, *SPTLC2*, *SPTLC2*, *SPTSSA*, *SGPP1*, *SPKH1*, *SPKH2*, *SGPL1* and *UGCG*. These results suggest that HexA inhibition promotes ganglioside biosynthesis (Figure 7D). These results contrast with the findings of Han et al., who reported downregulation of genes involved in ganglioside metabolism in fetal brain tissue from fetuses with confirmed TSD probably as a compensatory transcriptomic mechanism to prevent further ganglioside accumulation due to HexA impairment [39].

Finally, Bifsha et al., 2007 reported the downregulation of the *UCHL1* gene, which encodes ubiquitin C-terminal hydrolase L1, an important mediator of the ubiquitin-dependent protein degradation pathway, in several LSD skin fibroblast cultures [48]. Evaluation of *UCHL1* expression showed no difference in U87MG B2-7 cells compared to the WT control. Other genes related to proteasomal activity did not show any clear downregulation pattern (Figure 7E).

## 3. Discussion

U87MG is an immortalized cell culture of neoplastic origin that share traits of primary astrocytes such as GFAP, Vimentin, and S100β expression; as well as similar morphology, cytoplasmatic projections, cell linage origin, and expression of Excitatory Amino Acid Transporters (EAAT) than human astrocytes [50,51].

Although the physiology of U87MG cells differs from that of primary astrocytes, several studies have demonstrated that they reproduce pathophysiological alterations associated with neurodegenerative disorders [28,52]. For instance, U87MG cells have been shown to internalize and degrade β-amyloid aggregates through endocytic and lysosomal pathways, thereby recapitulating a critical mechanism of astrocytic clearance relevant to Alzheimer’s disease [52]. In addition, CRISPR/Cas9-mediated editing of GBA1 in U87MG cells induces key pathological changes, including inflammasome activation, α-synuclein aggregation, interleukin-1β production, and increased cell death, consistent with alterations reported in patients with Gaucher disease [28,31,53]. A recent work explored the bioactivity of pomegranate peel in an in-vitro Alzheimer’s disease model using U87MG cells and found reduced Aβ-amyloid aggregation and increased cell viability [29]. Taken together, this evidence supports the use of U87MG cells as a simplified yet informative model for investigating pathophysiological processes underlying neurodegenerative diseases, while also providing a practical platform for the screening of potential therapeutic strategies.

We aimed to develop an isogenic model of TSD that recapitulates key pathological features of LSDs, including lysosomal enlargement caused by the accumulation of partially degraded material, increased ROS production, and impaired autophagic flux. In addition, we sought to characterize the transcriptomic changes associated with HexA deficiency in astrocyte-like cells, as astrocytes play essential roles in CNS homeostasis and are increasingly recognized as contributors to the chronic neuroinflammation observed in gangliosidoses [26].

The CRISPR/Cas9-mediated disruption of *HEXA* in U87MG cells generated a cellular model that recapitulates the expected cellular alterations observed in TSD and other LSDs, including lysosomal enlargement, lipid accumulation, oxidative stress, and widespread transcriptional alterations. As expected, the absence of functional HexA activity resulted in the accumulation of undegraded lipidic material, leading to increased lysosomal content and disruption of lysosomal homeostasis. Beyond substrate storage, HexA deficiency was associated with a reduction in the activity of multiple lysosomal hydrolases and the transcriptional repression of genes involved in lysosomal biogenesis and function.

Although GM2 ganglioside is the primary substrate that accumulates in TSD, lysosomal dysfunction frequently affects the turnover of additional macromolecules [1]. The increase in GAGs levels therefore reflect secondary alterations in lysosomal homeostasis rather than a direct consequence of HexA deficiency. Impaired lysosomal function has been proposed to disrupt the coordinated degradation of multiple substrates [54]. Although all edited clones exhibited markedly reduced HexA activity, only two showed a significant increase in total GAGs levels. One possible explanation is that subtle differences in residual HexA activity among the clones, even within the low activity range, may be sufficient to differentially affect substrate turnover. In LSDs, residual enzyme activity is a major determinant of substrate accumulation and disease severity, as small variations around a critical functional threshold can produce disproportionately large differences in lysosomal storage [55]. Consequently, clones retaining residual HexA activity above this threshold may preserve sufficient degradative capacity to limit GAGs accumulation, whereas those with lower residual activity may be more susceptible to secondary substrate storage. Although residual HexA activity alone is unlikely to fully explain the observed heterogeneity, these findings suggest that relatively small differences in enzyme function may contribute substantially to the clone-dependent variability observed in our model.

The reduction tendency in the activity of multiple lysosomal hydrolases observed in U87MG B2-7 cells is consistent with the transcriptomic repression of genes involved in lysosomal biogenesis and function. Rather than representing a compensatory enhancement of lysosomal activity, these findings suggest a progressive deterioration of lysosomal homeostasis, in which HexA deficiency impairs the expression of genes required for lysosomal maintenance, thereby exacerbating substrate storage and cellular dysfunction. A previous study reported decreased expression of genes involved in lysosomal assembly and biogenesis in fetal brains from TSD patients [40], which is consistent with our findings and supports the notion that GM2 accumulation impairs lysosome biogenesis.

Mitochondrial dysfunction is a common consequence of impaired lysosomal homeostasis since nonfunctional mitochondria are normally recycled through mitophagy process which involves proper lysosomal function [37]. Due to mitophagy inhibition, aged mitochondria begin to accumulate in the cell, resulting in the alteration of normal equilibrium of mitochondrial biogenesis and recycling. The decrease in mitochondrial mass observed in *HEXA*-deficient cells contrasts with previous observations in TSD fibroblasts, highlighting the existence of cell-type-specific responses to lysosomal dysfunction. Transcriptomic analysis revealed significant downregulation of *PPARGC1A* (log_10_Fold Change = −0.94, False Discovery Rate = 4.8 × 10^−6^), a master regulator of mitochondrial biogenesis, together with marked induction of *KLF2* (log_10_Fold Change = 1.66, False Discovery Rate = 5.6 × 10^−14^). These findings suggest that impaired lysosomal homeostasis may actively repress mitochondrial biogenesis through a *KLF2–PPARGC1A* regulatory axis, providing a mechanistic explanation for the reduced mitochondrial content observed in the U87MG B2-7 model. This hypothesis is supported by a previous study which proposed a transcriptional mechanism by which the induction of the *KLF2* transcription factor represses mitochondrial biogenesis in Niemmann-Pick fibroblasts [56]. The same study reported increased mitochondrial mass in Niemann–Pick fibroblasts despite downregulation of mitochondrial genes, suggesting inefficient clearance of dysfunctional mitochondria combined with reduced biogenesis [56]. This finding is consistent with our observations in TSD fibroblasts (Supplementary Figure S6) and indicates that mitochondrial homeostasis may vary depending on the cell type. In our model, we observed reduced mitochondrial mass, further supporting the notion of cell type–specific alterations in mitochondrial regulation^74^. Overall, these results suggest that HexA deficiency has a deep effect on the transcription of genes associated with mitochondria biogenesis. This could be a countermeasure to prevent further damage of accumulated malfunctioning mitochondria. Lack of increase in mitochondrial mass could be explained by functional mitophagy in our *HEXA* KO model. In this regard, Matsushita et al. reported a small change in the expression of autophagy markers (LC3 and p62) in neuron progenitor cells derived from TSD iPSCs [25]. Altogether this data suggests that there is no strong mitophagy inhibition in our U87MG TSD model, although we observed elevated mitochondrial ROS that are not produced by altered mitochondrial membrane potential. In line with this conclusion, we observed reduced *SOD2* expression in U87MG B2-7, which could explain the increased mitochondrial ROS production without membrane potential disruption. Consistent with the increased mitochondrial and cellular reactive oxygen species, transcriptomic analysis further supports this interpretation revealing increased expression of the mitophagy receptors *BNIP3* (logFC = +0.53, False Discovery Rate = 1.05 × 10^−11^) and *BNIP3L* (logFC = +0.70, False Discovery Rate = 2.39 × 10^−15^) [57]. These findings suggest that chronic GM2 accumulation may promote adaptive remodeling of the mitochondrial network by simultaneously limiting mitochondrial biogenesis and enhancing selective mitochondrial turnover, thereby reducing mitochondrial mass while preserving the functional integrity of the remaining organelles. *HEXA* deficiency could be associated with mitochondrial alterations, however, the intrinsic metabolic characteristics of the U87MG glioblastoma cell line may influence mitochondrial function and bioenergetics. Therefore, validation of these findings in more physiologically relevant models, such as patient-derived induced pluripotent stem cell (iPSC)-derived neural cells, will be important to determine whether the mitochondrial alterations observed here accurately reflect the mechanisms operating in TSD.

Typically, LSDs disrupt the normal functioning of different organelles [58]. A previous study reported that the brains of a GM1 murine model exhibited elevated levels of ER vesicles and an increased ER-to-mitochondria ratio, highlighting organelle remodeling as a pathological feature of lysosomal storage disorders [59]. This is indicative of the natural progression of the disease occurring over the ER homeostasis. It has been reported that GM2 ganglioside can escape the lysosomes and interacts with the membranes of other organelles [1]. Accumulation of GM2 and other sphingolipids may disrupt membrane composition beyond the lysosomal compartment, affecting ER structure and function. Consistent with this possibility, HexA deficient cells displayed increased ER mass, which may initially represent an adaptive response aimed at preserving proteostasis. However, the reduced expression of *XBP1*, *ATF4*, and *DDIT3* suggests that prolonged lipid imbalance eventually compromises the ability of cells to mount an effective unfolded protein response, thereby contributing to chronic ER dysfunction. An alternative explanation of why HexA deficient cells exhibited increased ER-mass could be related to the change in gene expression of proteins related to ganglioside biosynthesis. Since glycosphingolipid biosynthesis originates in the ER before continuing through the Golgi apparatus, disturbances in ganglioside metabolism may increase the demand for lipid processing and membrane remodeling, thereby promoting structural adaptation of the endomembrane system. Notably, this interpretation is consistent with the absence of transcriptional activation of canonical unfolded protein response (UPR) genes, suggesting that ER expansion in HexA deficient cells may reflect metabolic and structural remodeling rather than a classical ER stress response.

Transcriptomic profiling of *HEXA*-deficient astrocyte-like cells revealed coordinated alterations in multiple biological processes, including extracellular matrix remodeling, developmental signaling, astrocyte-associated transcriptional programs, and gene sets associated with neuronal maturation and synapse organization. To independently validate these findings, we performed RT-qPCR analysis of six representative differentially expressed genes (*TIMP3*, *SALL1*, *PDPN*, *CCN1*, *GREM1*, and *CHRDL1*), selected because they encompass these major biological processes. The concordance between RT-qPCR and RNA-seq results supports the robustness of the transcriptomic dataset and indicates that the observed changes reflect coordinated remodeling of astrocyte molecular programs rather than isolated transcriptional events. In particular, the altered expression of genes involved in extracellular matrix regulation (*TIMP3*, *CCN1*, and *PDPN*) [60,61,62], astrocyte identity (*SALL1*) [63], developmental signaling (*GREM1*) [64], and neuronal maturation-associated pathways (*CHRDL1*) [65] suggests that HexA deficiency induces widespread transcriptional adaptations in astrocyte-like cells. While these findings do not directly demonstrate functional alterations in neuron–glia communication or synaptic organization, they identify molecular pathways that may contribute to disease pathogenesis and provide a framework for future studies using more physiologically relevant models, such as neuron–astrocyte co-culture systems.

Beyond alterations in specific biological pathways, our transcriptomic analysis suggests that HexA deficiency induces a broader transcriptional reprogramming through the dysregulation of key transcription factors, including *MYC* (logFC = +1.18, False Discovery Rate = 5.34 × 10^−21^), *SREBF2* (logFC = −0.51, False Discovery Rate = 2.34 × 10^−10^), and *XBP1* (logFC = −0.99, False Discovery Rate = 2.63 × 10^−16^). Because these regulators coordinate diverse processes, including lipid metabolism, membrane biogenesis, intracellular trafficking, and stress adaptation, their coordinated dysregulation provides a plausible mechanism linking GM2 accumulation to the widespread transcriptional remodeling observed in our model. These changes may therefore connect lysosomal GM2 storage with the alterations in glycosphingolipid metabolism, endomembrane organization, and other cellular processes identified in HexA deficient cells.

Functional enrichment analyses further revealed alterations in gene sets associated with neuronal maturation and synapse assembly. Astrocytes are known to contribute to synaptic development and maintenance through the expression of genes such as *GLUL*, *SLC1A2*, *SLC1A3*, and members of the *THBS* family. Therefore, the enrichment of these pathways in HexA deficient astrocyte-like cells suggests that transcriptional programs involved in neuron–glia communication are altered. Although these findings do not demonstrate functional impairment of synapses or neuronal activity, they raise the possibility that HexA deficiency may influence astrocyte-mediated support of neuronal homeostasis. Future studies using neuron–astrocyte co-culture systems or other functional models will be required to determine whether these transcriptomic alterations translate into measurable effects on synaptic organization and neuronal function.

**Figure 8:**
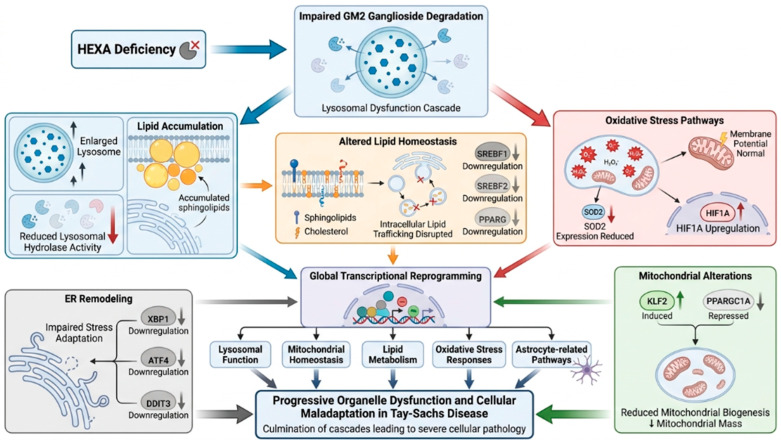
Proposed mechanistic model of HEXA deficiency in Tay–Sachs disease U87MG cellular model. HEXA deficiency leads to GM2 ganglioside accumulation and lysosomal dysfunction, characterized by lysosomal enlargement, lipid accumulation, and reduced hydrolase activity. Altered lipid homeostasis affects intracellular trafficking and transcriptional regulation (e.g., SREBF1/2, PPARG). Lysosomal stress promotes oxidative imbalance and pseudohypoxic adaptation, along with reduced mitochondrial biogenesis and mass. Concurrent changes in lipid metabolism contribute to ER remodeling and impaired stress responses. Together, these alterations drive coordinated transcriptional reprogramming that disrupts organelle function and cellular homeostasis. Image was generated by using https://www.figurelabs.ai/ (access 30 June 2026).

Taken together, the cell model here described reproduces the genetic and enzymatic defects associated with Tay–Sachs disease. Our results support a mechanistic model in which *HEXA* deficiency initiates a cascade of interconnected cellular alterations that extend beyond the primary accumulation of GM2 gangliosides (Figure 8). The impaired degradation of GM2 disrupts lysosomal homeostasis, leading to lysosomal enlargement, lipid accumulation, and secondary reduction of multiple lysosomal hydrolase activities. Accumulated sphingolipids may alter membrane composition and intracellular lipid trafficking, which is consistent with the downregulation of the lipid-sensing transcription factors *SREBF1*, *SREBF2*, and *PPARG*. In parallel, lysosomal dysfunction promotes oxidative stress, as evidenced by increased total and mitochondrial ROS, while the absence of major changes in mitochondrial membrane potential suggests that oxidative imbalance is not primarily driven by mitochondrial depolarization. Instead, the reduced expression of *SOD2* may contribute to superoxide accumulation, whereas the upregulation of *HIF1A* indicates activation of a pseudohypoxic metabolic adaptation. Furthermore, the concomitant induction of KLF2 and repression of *PPARGC1A* support a transcriptional mechanism linking lysosomal stress to reduced mitochondrial biogenesis, thereby explaining the decrease in mitochondrial mass observed in *HEXA*-deficient cells. Altered lipid homeostasis may also affect endoplasmic reticulum membrane organization, contributing to ER remodeling and compromised stress adaptation, which is reflected by the downregulation of the UPR-associated transcription factors *XBP1*, *ATF4*, and *DDIT3*. Collectively, these alterations converge into a broad transcriptional reprogramming that impacts lysosomal function, mitochondrial homeostasis, lipid metabolism, oxidative stress responses, and astrocyte-related pathways, ultimately providing a unified model in which chronic GM2 accumulation drives progressive organelle dysfunction and cellular maladaptation in TSD. Finally, although the U87MG-*HEXA* knockout model does not fully recapitulate the cellular complexity of the human brain or the phenotypic diversity of TSD, it represents a valuable platform for mechanistic discovery. The controlled genetic background and experimental accessibility of this model enable the systematic investigation of molecular pathways that are altered as a direct consequence of HexA deficiency. Such an approach facilitates the identification of early cellular responses and probable pathogenic mechanisms that may otherwise be difficult to dissect in more complex experimental systems. Importantly, the molecular alterations identified in this model provide valuable mechanistic insights into the cellular consequences of HexA deficiency and establish a foundation for further investigation in more physiologically relevant experimental systems. Mechanisms identified in this simplified cellular model can subsequently be validated in more physiologically relevant systems, including patient-derived iPSC-based neural models and genetically engineered mouse models, thereby providing a complementary strategy to unravel the molecular basis of TSD.

## 4. Materials and Methods

### 4.1. Cell Cultures

U87MG (ATCC^®^ HTB-14™) human glioblastoma cell line was obtained from the American Type Culture Collection (ATCC, Manassas, VA, USA) and were maintained in DMEM medium, 15% fetal bovine serum, 1% penicillin/streptomycin. Skin fibroblasts derived from both healthy (GM23963C) and Tay-Sachs individuals (GM00515B, Coriell Institute, Camden, NJ, USA) were maintained in DMEM medium, 15% fetal bovine serum, 1% penicillin/streptomycin. HEK 293 FT cells were maintained in DMEM medium, 10% fetal bovine serum, and 1% penicillin/streptomycin. All cell cultures were incubated in a humidified environment at 37 °C, CO_2_ 5%.

### 4.2. Construction of CRISPR-Cas9/sgRNA HEXA Vectors

The CRISPR-ERA [66], CHOP-CHOP [67], and IDT (https://www.idtdna.com/page/products/crispr-genome-editing, access 1 September 2022) were used to design a sgRNA to direct the Cas9 system to the *HEXA* gene *locus*. One sgRNA was selected from each of these tools that targeted the first exon of the *HEXA* gene (Supplementary Figure S1) and had the highest score in terms of cutting efficiency and the lowest probability of off-target effects (Supplementary Table S1). The sgRNAs were synthetized and cloned into Addgene 64324 plasmid (hereafter CRISPR-Cas9 plasmid) as previously described [68] (Supplementary Figure S2A,B). This plasmid encodes for *Stapylococcus pyogenes* Cas9 (Protospacer Adjacent Motif: NNG) under the control of cytomegalovirus promoter, and it has a mCherry reporter gene. Briefly, the CRISPR-Cas9 plasmid was digested with *BbsI*-HF (New England Biolabs, Ipswich, MA, USA, cat no. R539S) and ligated with the sgRNAs using T4 DNA ligase kit (New England Biolabs, Ipswich, MA, USA, cat no. M0202S). Insertion of the sgRNAs was verified by Sanger sequencing (Supplementary Figure S2C).

### 4.3. Off-Target Effects Prediction

Designed sgRNA sequences were submitted to the CRISPOR [69] online tool (https://crispor.gi.ucsc.edu/, 1 September 2022) for off-target prediction using the human genome GRCh38 as template. The Cutting Frequency Determination (CFD) was estimated for each predicted off-target site. The CFD score represents the predicted relative cleavage efficiency of each off-target site compared with a perfectly matched on-target sequence [70]. CFD values below 0.02 indicate very low cleavage efficiency compared with the on-target sequence, whereas a CFD value of 1 reflects high cleavage efficiency at an off-target site (Supplementary Table S1).

### 4.4. T7 Endonuclease I Mismatch Detection Assay

To confirm that the sgRNAs were able to induce mutagenesis on the *HEXA* gene locus, we first transfected HEK 293FT cells with CRISPR-Cas9 plasmid with or without sgRNA sequence using Lipofectamine 3000 (Thermo Fisher Scientific, Waltham, MA, USA; catalog no. L3000015) according to manufacturer instructions. Three days after transfection, genomic DNA was extracted using the Monarch Genomic DNA Purification Kit (New England Biolabs, Ipswich, MA, USA, cat no. T3010S). A 1 kb fragment was PCR amplified (Forward: TACTTCAGCCTGGCAAGTCCTT, and Reverse: GCCCTTGCTCACAGTCTCACTA) (Supplementary Table S2) and incubated with a T7 endonuclease I from the EnGen^®^ Mutation Detection Kit (New England Biolabs, Ipswich, MA, USA, cat no. E3321S). The cutting percentage was calculated by densitometry on a 1% agarose gel using GelAnalyzer 19.1 software (www.gelanalyzer.com, 1 February 2023).

### 4.5. Generation of Hexa Deficient Model from U87MG Lines

U87MG cells were seeded at a density of 100,000 cells/well in a 12-well plate (TPP, Sigma-Aldrich, St. Louis, MO, USA; Product 92012). 500 ng of the CRISPR-Cas9/sg*HEXA* plasmid with the best T7 endonuclease I result, was transfected using Lipofectamine 3000 (Thermo Fisher Scientific, Waltham, MA, USA; catalog No. L3000015) according to manufacturer instructions. Cells with positive mCherry fluorescence were isolated by cell sorting using the BD FACSAria II cell sorter (Becton Dickinson, Franklin Lakes, NJ, USA) using the PerCP-CY5-5-A filter. Single clones were grown until confluence, and those that present reduced or no HexA enzymatic activity were selected for subsequent sequencing and evaluation of biomarkers related to TSD.

### 4.6. Indel Detection by Chromatogram Decomposition Analysis

From different cell populations obtained from single cell isolations that showed reduced HexA enzymatic activity, genomic DNA was extracted as previously described. A 700 bp region of the *HEXA* first exon containing the cutting site was amplified by using the Q5^®^ High-Fidelity DNA Polymerase (New England Biolabs, Ipswich, MA, USA, cat no. M0515) with the same primers used in the T7 assay. The PCR fragments were purified using the Monarch^®^ Genomic DNA Purification Kit (New England Biolabs, Ipswich, MA, USA, cat no. T3010S) and sequenced by the Sanger method (Macrogen, Seoul, Republic of Korea). The resulting chromatograms were analyzed using TIDE (Tracking of Indels by DEcomposition) bioinformatics tool, as previously described [71,72].

### 4.7. Enzymatic Activity

Specific enzymatic activity of five different lysosomal hydrolases was evaluated: HexA, N-acetylgalactosamine-6-sulfatase (GALNS), β-Galactosidase (β-Gal), β-Glucoronidase (GUSB) and N-acetyl-glucosaminidase (NAGLU). Cells were seeded at 100,000 cells/well in 6-well plate (TPP, Sigma-Aldrich, St. Louis, MO, USA; Product 92006). Cell lysates were obtained by treating the cells with 25 mM citrate-sodium buffer (pH 4.5, 0.5% Triton X-100). After 30 min of incubation at 4 °C, the supernatant was removed and placed on 1.5 mL tubes for later centrifugation at 5000 rpm for 5 min. Supernatant were translated into new 1.5 mL tubes. All lysates were kept at −20 °C until enzymatic activity measurement. All samples were stored for no longer than one week before enzymatic activity was measured. The enzymatic activity for each enzyme was measured as follows:

- **HexA**: 50 µL of cell lysate were mixed with 20 µL of 3.2 µM of 4-methylumbelliferyl-β-D-N-acetyl glucosamine-6-sulfate substrate (MUGS) (EMD Millipore Corp., Burlington, MA, USA) previously resuspended on 0.1 M sodium citrate (pH 4.4). The reaction was incubated for 20 min at 37 °C and then stopped with 150 µL of stop buffer (0.17 M C_2_H_5_NO_2_/NaHCO_3_ pH 9.8).
- **GALNS**: 10 μL of cell lysate were mixed with 20 μL of 2 mM 4-methylumbelliferyl-β-D-galactopyranoside-6-sulfate substrate (Toronto Chemicals Research, North York, ON, Canada) previously resuspended 0.1 M sodium acetate (pH 4.3) and incubated for 17 h at 37 °C. 2 µL of β-galactosidase from *Aspergillus oryzae* (10 mg/mL) were added and the reaction was incubated for 2 h at 37 °C. The reaction was stopped with 150 µL stop buffer.
- **β-Gal**: 20 µL of cell lysate were mixed with 30 µL of 2 mM 4-methylumbelliferyl-β-D-galactopyranoside substrate (Sigma-Aldrich, St. Louis, MO, USA: CAS 6160-78-7) previously resuspended in 0.1 M citrate-phosphate (pH 4.3). The reaction was incubated for 3 h at 37 °C and then stopped with 150 µL stop buffer.
- **GUSB**: 50 µL of total cell lysate were mixed with 50 µL of 1 mM 4-Methylumbelliferyl-β-D-glucuronide substrate (Sigma-Aldrich, St. Louis, MO, USA: Cat. No. M-3633) previously resuspended in 0.1 M sodium acetate (pH 4.5) and incubated 24 h at 37 °C. The reaction was stopped with 150 µL stop buffer.
- **NAGLU**: 50 µL of total cell lysate were mixed with 50 µL of 2 mM 4-Methylumbelliferyl N-acetyl-α-D-glucosaminide (4-MU-α-GlcNAc) (Sigma-Aldrich, St. Louis, MO, USA: CAS 80265-04-9) previously resuspended in ultrapure water and incubated 1 h at 37 °C. The reaction was stopped with 150 µL stop buffer.

For all enzymatic reactions, fluorescence was measured in a Twinkle LB 970 fluorescence microplate reader excitation/emission: 360 nm/445 nm (Berthold Technologies Microplate). A standard curve was made by using serial dilutions from a solution of 2 µM 4-methyl-umbelliferone. Finally, specific enzymatic activity was expressed as U/mg of total protein determined using the BCA Protein Assay Kit (Thermo Fisher Scientific, Waltham, MA, USA; cat no. 23227). A total of 10–20 µg of protein was used for each enzymatic activity assay. One unit of enzymatic activity was defined as the amount of enzyme required to hydrolyze 1 nmol of synthetic substrate per hour.

### 4.8. Lysosomal Mass Evaluation

Lysosomal mass evaluation was carried out as previously reported [38,71]. Cells were seeded at confluency of 100,000 cell/well in a 6-well plate (TPP, Sigma-Aldrich, St. Louis, MO, USA; Product 92006), one day before the lysosomal mass evaluation. Cells were incubated at 37 °C, 5% CO_2_ with 50 nM Lysotracker Deep Red (Thermo Fisher Scientific, Waltham, MA, USA; cat. no. L12492). After 1 h, cells were trypsinized and resuspended in Hank’s Buffered Salt Solution (HBSS) buffer with 1 µg/mL propidium iodide. Cells were analyzed using a BD FACSAria II cell sorter (Becton Dickinson, Franklin Lakes, NJ, USA) using PerCP filter (Ex: 476 nm/Em: 680 nm). Mean fluorescence from events of three independent replicates were analyzed using the FlowJo software (FlowJo vX.0.7). For visualization by epifluorescence microscopy, cells were seeded in 14 mm cover slips, incubated with 50 nM Lysotracker Deep Red, washed with 1X PBS, incubated with PFA 4% for 10 min, and mounted on slides using Gold Antifade Mountant medium (Thermo Fisher Scientific, Waltham, MA, USA; cat. no. P36930). Images were obtained on a Zeiss AXIO observer Z1 microscope (Zeiss, Jena, Germany) and analyzed using the Image J software 1.54t (http://imagej.org, accessed on 14 May 2026).

### 4.9. Mitotracker Green FM Staining

MitoTracker reagents are fluorescent dyes that selectively label mitochondria in live cells allowing for visualization of their structure, number and activity. Specifically, MitoTracker Green FM label mitochondria independently of its membrane potential state [73]. Therefore, it can be used for mitochondrial mass quantification. Cells were seeded at 100,000 cells/well in a 6 well plate and stained with MitoTracker Green FM fluorescent dye (Thermo Fisher Scientific, Waltham, MA, USA; cat. no. M7514) according to manufacturer protocol. Briefly, 100 nM of MitoTracker Green FM were incubated on live cells for 30 min. Then cells were trypsinized and washed with 1X PBS twice. Cells were centrifugated at 1600 rpm for 10 min and resuspended on HBSS buffer. Cells were analyzed on a BD FACSAria II cell sorter (Becton Dickinson, Franklin Lakes, NJ, USA) using the FITC filter (Ex: 475 nm/Em: 530 nm). Mean fluorescence from events of three independent replicates were analyzed using the FlowJo software (FlowJo vX.0.7). For live cell visualization, cells were seeded at 25,000 cells/well in a 24 well plate and incubated with MitoTracker Green FM as previously described. The media with Mitotracker Green FM was removed and replaced with HBSS Buffer with 5 µg/mL of Hoechst 33342 for 10 min and visualized on a Zeiss AXIO observer Z1 microscope (Zeiss, Jena, Germany). Images were analyzed using the Image J software 1.54t (http://imagej.org, accessed on 14 May 2026).

### 4.10. Reactive Oxygen Species Evaluation (ROS)

Cells were seeded at confluence of 100,000 cell/well in a 6 well plate a day before ROS evaluation. Before staining, positive controls were incubated with 500 nM (U87MG) or 100 nM (skin fibroblasts) of rotenone (Sigma-Aldrich, St. Louis, MO, USA; cat. no. R8875) for 16 h. Cells were resuspended in HBSS buffer with 1 µM 2′, 7′-dichlorodihydrofluorescein diacetate (H_2_DCFDA, Thermo Fisher Scientific, Waltham, MA, USA, cat. no. D399). After incubation for 30 min at 37 °C, cells were washed and resuspended with HBSS buffer with 1 µg/mL of propidium iodide to evaluate cell viability. Cells were analyzed on the BD FACSAria II cell sorter (Becton Dickinson, Franklin Lakes, NJ, USA) using the FITC filter (Ex: 475 nm/Em: 530 nm). Mean fluorescence from events of three independent replicates were analyzed using the FlowJo software (FlowJo vX.0.7).

### 4.11. Mitochondrial Reactive Oxygen Species Evaluation

Superoxide anion is considered an indicator of mitochondrial derived ROS and subsequently is an indicator of the mitochondrial state. MitoSox Red (Thermo Fisher Scientific, Waltham, MA, USA; cat. no. M36008) is selectively oxidized by mitochondrial superoxide [74]. To quantify the contribution of mitochondria on the oxidative state, the cells were seeded at concentration of 100,000/well in a 6 well plate. The cells were incubated with 1 µM MitoSox Red in HBSS buffer and cells were incubated for 10 min. After incubation, cells were detached, centrifugated at 1600 rpm for 10 min, and resuspended on fresh HBSS buffer. Cells were analyzed on a BD FACSAria II cell sorter (Becton Dickinson, Franklin Lakes, NJ, USA) using the Texas Red filter (excitation: 560/40 nm, emission: 630/75 nm). Mean fluorescence from events of three independent replicates were analyzed using the FlowJo software (FlowJo vX.0.7).

### 4.12. Mitochondrial Membrane Potential Evaluation

To measure differences in mitochondria membrane potential, cells were seeded at confluency of 10,000 cells/well in a 12-well plate. Cells were trypsinized and resuspended in 1X PBS with 2.5 µg/mL JC-1 (Thermo Fisher Scientific, Waltham, MA, USA; cat. no. T3168). After 10 min at 37 °C, cells were centrifugated 5 min at 1600 rpm and resuspended in 1x PBS. Samples were analyzed on a BD FACSAria II cell sorter using FITC filter (Ex: 475 nm/Em: 530 nm) and PE filter (Ex: 488/Em: 578 nm). Mean fluorescence from events of three independent replicates were analyzed using the FlowJo software (FlowJo vX.0.7). For positive controls, cells were then incubated with rotenone (Sigma-Aldrich, St. Louis, MO, USA, cat. no. R8875) at final concentration of 500 nM for U87MG cells and 100 nM for fibroblast at 37 °C, 5% CO_2_. After 18 h of incubation, cells were trypsinized and treated with JC-1 as described above.

### 4.13. ER-Tracker Quantification

ER-tracker is a cell-permeant fluorescence dye that binds to ATP sensitive to K^+^ channels which are abundant on endoplasmic reticulum (ER) membranes [75]. Cells were seeded at 50,000 cells/well in a 12-well plate (TPP, Sigma-Aldrich, St. Louis, MO, USA; Product 92012). The day after, cells were incubated with 1 µM Thapsigargin (Sigma-Aldrich, St. Louis, MO, USA, cat. no. T9033) during 15 h at 37 °C, 5%CO_2_ as positive control of prolonged ER-Stress [76]. All cells were incubated with 1 µM ER-Tracker Green (Thermo Fisher Scientific, Waltham, MA, USA, cat. no. E34250) for 30 min at 37 °C, 5% CO_2_. Cells were then trypsinized, centrifugated at 1600 rpm for 10 min and resuspended on HBSS buffer. All samples were analyzed with a BD FACSAria II cell sorter (Becton Dickinson, Franklin Lakes, NJ, USA) using FITC filter (Ex: 475 nm/Em: 530 nm). Mean fluorescence from events of three independent replicates were analyzed using the FlowJo software (FlowJo vX.0.7).

### 4.14. Evaluation of Neutral Lipids

Cells were seeded at confluency of 100,000 cell/well in a 6-well plate a day before Nile Red evaluation. Cells were trypsinized and resuspended in HBSS buffer with 1 µM Nile Red (Sigma-Aldrich, St. Louis, MO, USA, cat. no. 72485). After 10 min of incubation, cells were washed and resuspended in a fresh HBSS buffer. Cells were analyzed on a BD FACSAria II cell sorter (Becton Dickinson, Franklin Lakes, NJ, USA) using FITC filter (Ex: 475 nm/Em: 530 nm). Mean fluorescence from events of three independent replicates were analyzed using the FlowJo software (FlowJo vX.0.7).

### 4.15. Determination of Total Glycosaminoglycans

For the determination of glycosaminoglycans (GAGs) in cell culture medium, the 1,9-dimethylmethylene blue (DMB) (Sigma-Aldrich) assay was used [38]. Briefly, 50 uL of cell lysate from each cell culture was incubated for 1 min with 275 uL of DMB at room temperature. Absorbance was read in a spectrophotometer Shimadzu UV-1700 (Shimadzu Corporation, Kyoto, Japan) at 520 nm during the first 5 min of reaction. The concentration of the GAGs was determined using a standard curve of chondroitin sulfate A (Thermo Fisher Scientific, Waltham, MA, USA, cat. no. J60341.06).

### 4.16. Transcriptome Assembly and Analysis

Total RNA was extracted by triplicate for each condition by a TRIzol-Chloroform protocol. Briefly, 150,000 cells were seeded on a 6-well plate by triplicate. After 5 days, cells were incubated with 1 mL of TRIzol reagent (Thermo Fisher Scientific, Waltham, MA, USA; cat. no. 15596026) for 5 min at room temperature. Then, 200 µL of chloroform were added and the solution was homogenized by agitation. After centrifugation at 12,400 rpm for 10 min at 4 °C, the aqueous phase was collected, and RNA was precipitated with 500 µL of isopropanol. The RNA was dried and resuspended on 50 µL DEPC treated water. Total RNA was sequenced on a NovaSeq X Plus Series platform (Illumina, San Diego, CA, USA) on a PE150 protocol after reverse transcription, second cDNA synthesis, end repairing and A-tailing (Novogene Corporation Inc., Sacramento, CA, USA). Raw FastQ files obtained after RNA sequencing are available at the Gene Expression Omnibus (GEO) database with Accession number GSE329156. Trimming and sequence alignments were conducted in a Conda environment manager installed in the ZINE HPC cluster at Pontificia Universidad Javeriana. Sequences were trimmed by using trimmomatic (0.40–0). Subsequently, the STAR 2.7.3a software [77] was implemented for sequence alignment using the reference genome GRCh38. Normalization and differential gene expressions were conducted using edgeR (4.6.3) from the Bioconductor repository. Gene ontology analysis on differentially expressed genes was done using the KEGG database [78], Reactome [79] and the DAVID online tool [80]. All images of gene expression analysis were created using the RStudio integrated development environment (R studio 2025.05.1 Build 513).

### 4.17. Rt-qPCR

Total RNA was isolated as described for transcriptome analysis. Subsequently, single strand cDNA was synthetized by reverse transcription PCR using a RevertAid First Strand cDNA Synthesis Kit (Thermo Fisher Scientific, Waltham, MA, USA; cat. no. K1622). The quality of cDNA samples was later verified by PCR. Different sets of primers targeting specific genes (Supplementary Table S2) were used for qPCR by using the Luna Universal qPCR master mix (2X, New England Biolabs, Ipswich, MA, USA) as indicated by manufacturer protocols.

### 4.18. Statistical Analysis

Statistical software R version 4.3.1 or Graph Prism 8.0.2 (GraphPad Software, Boston, MA, USA) was used to conduct parametric or non-parametric ANOVA or t-student tests where necessary. For non-parametric ANOVA, Statistical significance was assessed using the Kruskal–Wallis test followed by Dunn’s multiple comparison test.

## 5. Conclusions

In this study, we have developed a model for TSD using an the U87MG cell line that holds some features of human astrocytes, which make it a relevant model for studying physiopathology mechanisms occurring in cells from the human glial cells in the context of TSD. The model recapitulates key features common in TSD and other LSDs. Aside from cellular changes in lysosomal mass, lipid content, mitochondrial state, and ER homeostasis, we observed a deep transcriptional shift leading to overexpression on genes related to neuronal maturation, stem cell maintenance, ganglioside metabolism, and mitochondrial and lysosomal biogenesis. These findings expand our knowledge regarding the alterations that occur in glia cells which are important for maintenance of the CNS homeostasis. Future efforts should be directed at studying how these alterations could contribute to the progressive neurodegeneration seen on TSD patients. Understanding the neuron-glia axis interaction could lead to the discovery of new alternative therapeutic targets that help us create new therapeutic strategies for TSD. Future studies using primary astrocytes, induced pluripotent stem cell (iPSC)-derived astrocytes, or animal models will be important to validate and extend the observations reported here.

## Data Availability

The data supporting this study are openly available at the Gene Expression Omnibus (GEO) database with Accession number GSE329156.

## Supplementary Materials

The following supporting information can be downloaded at: https://www.mdpi.com/article/10.3390/ijms27146503/s1.
