# Supplementary material for: Modeling Tay-Sachs Disease in Astrocyte-like Cells Reveals Significant Changes in the Transcriptomic Profile
Source: Int J Mol Sci. 2026 Jul 22;27(14):6503. doi: 10.3390/ijms27146503 (PMC13411418; doi:10.3390/ijms27146503)
Supplement: Supplementary file 1 [file ijms-27-06503-s001.zip › ijms-4373766-supplementary.pdf]

Supplementary Information

Modeling Tay-Sachs Disease in Astrocyte-Like Cells Reveals Significant Changes in the Transcriptomic Profile

Diego A. Suárez-Garcia[1], Angela J. Espejo-Mojica[1], Carlos J. Alméciga-Díaz[1, *]

[1] Institute for the Study of Inborn Errors of Metabolism, Faculty of Science, Pontificia Universidad Javeriana, Bogotá D.C, Colombia

* Corresponding author
Carlos J. Alméciga-Díaz, BPharm, PhD. Institute for the Study of Inborn Errors of Metabolism, Faculty of Science, Pontificia Universidad Javeriana, Cra 7 # 42-46, Edificio Felix Restrepo S.J., Bogotá D.C, Colombia, 110231.

Supplementary Figure S1. Genomic sequence of the first exon from the *HEXA* gene indicating the targeted sites of the three designed sgRNAs. The image was obtained from Benchling (2026). Retrieved from https://benchling.com... 4

Supplementary Table S1. Predicted off-target sites of the selected sgRNA sequences designed to target exon 1 of the HEXA gene on chromosome 15 based on results from the CRISPOR online tool. Red letters indicated mismatched nucleotides on the DNA sequence. sgRNA1, sgRNA2 and sgRNA3sequences were designed using CRISPR-ERA, IDT design tool and CHO-CHOP web tool respectively. Cutting Frequency Determination (CFD) was predicted for each potential off-target site……………………………………………………………………………………………….……5

Supplementary Figure S2. (A) CRISPR-Cas9 plasmid holding U6 promoter for sgRNA expression, sgRNA scaffold for roper association with the Cas9 enzyme, Cytomegalovirus enhancer (CMV Enhancer), a chimeric intron, a SV40 nuclear localization signal (SV40 NLS), Streptococcus pyogenes Cas9 sequence, mCherry reporter sequence, Ampicillin resistance factor (AmpR) and an origin replication site (Ori). Plasmid sequence can be found on the Addgene repository (Addgene # 64324). (B) Image summarizing sgRNA cloning into the CRISPR-Cas9 plasmid by enzymatic digestion using a BbsI endonuclease for later purification of the linearized plasmid and cloning of sgRNAs by enzymatic ligation. (C) Molecular confirmation of sgRNAs sequence by Sanger sequencing. Three sgRNAs were independently cloned into CRISPR-Cas9 plasmid. The predicted sequence of the plasmids holding sgRNA sequence (Top of each alignment) completely align with the sanger sequences. Highlighted letters indicate sgRNA sequences. ……….….6

Supplementary Table S2. List of primers used in this study for transcriptome validation, evaluation of Endoplasmic reticulum (ER) Stress, assemble, molecular verification of CRISPR-Cas9/sgRNAs plasmids, and T7 mismatch assay….7

Supplementary Figure S3. U87MG cells were transfected with the CRISPR-Cas9/sgRNA holding the highest cutting efficiency, sorted by FACS and diluted to a density of 1 cell/well for population expansion and subsequent selection of knocked-out clones……………………………………………………………………………………..….….….8

Supplementary Figure S4. Enzymatic HexA measurement and Total GAGs quantification on three U87MG clones after single cell isolation of cells transfected with CRISPR-Cas9/sgRNA *HEXA* plasmid. **(A)** Enzymatic hex-A activity was measured on three B2-2, B2-16 and B2-19 clones isolated from U87MG cell culture transfected with CRISPR-Cas9/sgRNA *HEXA* plasmid. **(B)** Total GAGs were measured from cell lysates of different Hex-A deficient clones. Data are presented as mean ± SEM. Statistical significance was assessed using the Kruskal-Wallis test (** $p < 0.005$) (* $p < 0.05$) followed by Dunn’s multiple comparison test……………………………………………………..………………9

Supplementary Figure S5. Predicted Indel proportion on the HEXA locus region sequence obtained from cells transfected with CRISPR-Cas9/sgRNA (Top). Predicted Indels with p<0,001 are shown with red bars. Indel detection analysis by Chromatogram decomposition of sequences from different cell populations (bottom). Chromatogram decomposition of sequences from cells transfected with CRISPR-Cas9 (Green lines) vs untreated control (Black bars). The dotted blue line shows the region where the CRISPR-Cas9 was predicted to cut the genomic HEXA locus. The larger the lines the more background noise is expected to be found on the genomic region indicating presence of Indels. Electropherograms of DNA sequences from (A) B2-2, (B) B2-7, (C) B2-16 and (D) B2-19 cell cultures were analyzed using the TIDE online tool (https://apps.datacurators.nl/tide/) 10

Supplementary Figure S6. HexA enzymatic activity was measured at different time points in lysates from four independent clones with confirmed *HEXA* knockout. Activity is expressed as a percentage of that measured in wild-type (WT) cells (WT = 100%) …………………………………………………………………………………………11

Supplementary Figure S7. (A) Enzymatic hex-A activity on unaffected skin fibroblasts and TSD fibroblasts. Enzymatic activity was obtained by 12 independent measurements. (B) Lysotracker Deep Red staining for Lysosomal mass visualization on epifluorescence microscopy (Left 20X) and quantification by flow cytometry (Right). (C) Cells labeled with MitoTracker green FM for mitochondria staining on epifluorescence microscopy (Left 20X) and quantification by flow cytometry (Right). *** $p < 0.001$. Mann-Whitney test. Fluorescence quantification by flow cytometry is derived from data of three independent experiments. **** $p < 0.0001$. Kolmogorov-Smirnov test…………………………………………12

Supplementary Figure S8. Oxidative stress evaluation on TSD skin fibroblasts. (A) H2DCFDA staining for ROS measurement on TSD skin fibroblasts by flow cytometry. Dot plots show increase on fluorescence intensity (Left). Histograms of positive stained cells (middle). Graph showing fluorescence distribution of each cell population (Right) (B) MitoSOX fluorescence intensity measurement on TSD skin fibroblasts by flow cytometry. Dot plots show findings on labeled cell with MitoSOX reagent (Left). Histograms of positive stained cells (middle). Graph showing fluorescence distribution of each cell population (Right) (C) Representative quadrant plots of non-labeled cells, cell treated with Rotenone (ROT), WT cells, and U87MG B2-7 clone. X axis represents FITC fluorescence and Y axis represent PE fluorescence. Fluorescence quantification by flow cytometry is derived from data of three independent experiments. **** $p < 0.0001$. Kolmogorov-Smirnov test or Kruskal-Wallis test (*<0.05)……………………………………………………13

Supplementary Figure S9. Relative gene expression of U87MG B2-7 cells compared to a WT control. Genes shown in the x axis are related to ER-stress. Gene expression is presented as Log10 Fold change (compared to control). Statistical significance in gene expression change is considered at FDR <0.05……………………………………………………...14

Supplementary Figure S10. (A) Quality scores from RNA samples extracted from U87MG WT and B2-7 clone. (B) Quality control boxplot of RNA-Seq counts after normalization by TMM method using edgeR library from R………………....15

Supplementary Figure S11. Gene ontology (GO) analysis of the 50 biological processes (BP) terms with genes having most variable expressions on U87MG B2-7 clone compared to the control (WT). (A) BP related to downregulated genes are shown on the left and BP related to upregulated genes are on the right (B). Gene ratios on x axis are the number of genes on each BP term divided by the total number of genes evaluated. Dots size represents the number of genes involved with each BP term while color represents p-values. P-values<0.05 are considered statistically significant…...16

Supplementary Figure S12. Dot plot enrichment analysis based on KEGG terms of downregulated (Left) and upregulated (Right) genes on U87MG B2-7 clone compared to the control. Gene ratios on x axis are the number of genes on each KEGG term divided by the total number of genes evaluated. Dots size represents the number of genes involved with each KEGG term while color is representative of p-value significance. P-values<0.05 are considered statistically significant……………………………………………………………………………………………………...…...17

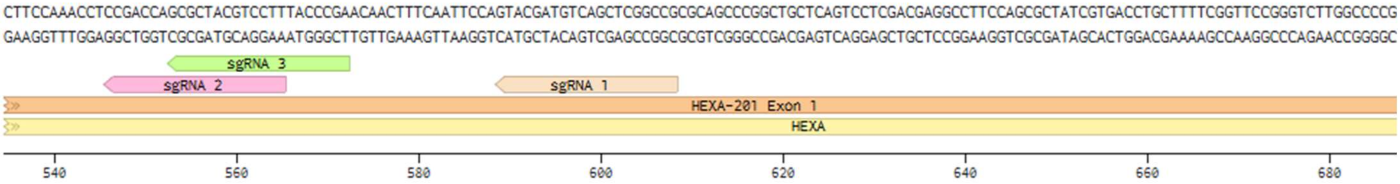

Supplementary Figure S1. Genomic sequence of the first exon from the *HEXA* gene indicating the targeted sites of the three designed sgRNAs. The image was obtained from Benchling [Biology Software]. (2026). Retrieved from https://benchling.com.

Supplementary Table S1. Predicted off-target sites of the selected sgRNA sequences designed to target exon 1 of the *HEXA* gene on chromosome 15 based on results from the CRISPOR online tool. Red letters indicated mismatched nucleotides on the DNA sequence. sgRNA1, sgRNA2 and sgRNA3sequences were designed using CRISPR-ERA, IDT design tool and CHO-CHOP web tool respectively. Cutting Frequency Determination (CFD) was predicted for each potential off-target site.

sgRNA 1: CGGCCGAGCTGACATCGTAC

Sequence 5´-3´	Chromosome	CFD off-target score
CAACGGAGCTGACATCGTAA	16	0.19
TGGCTGAGCTGATATCCTAC	X	0.10
CGGGGGAGCTGACATAGGAC	11	0.10
CAGCCCAGCTGACATCTTAA	3	0.07
CTGCCCTGCTGACATTGTAC	12	0.06
GGGCCGGGCTGCCATCGGAC	19	0.06
CTGCCCTGCTGACACCGTAC	3	0.05
CGGCGGAGCTGCCTTCGTTC	17	0.05
CGGCCAAGGTGACATTCTAC	19	0.04
GGGCCGAGCTGGCATCCTCC	9	0.03

sgRNA 2: AAGGACGTAGCGCTGGTCGG

Sequence 5´-3´	Chromosome	CFD off-target score
AAGAAAGGAACGCTGGTCGG	19	0.57
AAGGACACAGAGCTGGTCAG	14	0.53
AAGGACACAGAGCTGGTAGG	19	0.40
AAGGACGCAGAGCTGGCCAG	16	0.38
AAGGACGTAGAACTGGACGG	3	0.37
AAGGACATAGAACTGGACGG	17	0.37
CAGGATGTAGAGCAGGTCGG	17	0.37
AAGGACGTGGAGCTGGTTGG	16	0.32
AATGACGTATAGCTGATCGG	12	0.30
AGGGAATTAGCGCTGGTCAG	11	0.30

sgRNA 3: TCGGGTAAAGGACGTAGCGC

Sequence 5´-3´	Chromosome	CFD off-target score
TGGGGAAAAGGAAGTAGCTC	16	0.15
CCTGCTCAAGGACGTAGCGC	20	0.15
GCGGGTAAAGGATTTAGCAC	5	0.13
TCAGGAAAAGGACTTAGCAC	21	0.12
TCGGGTAAGTGACTTAGCTC	12	0.10
GCTGGTAAAGGTCGAAGCGC	7	0.10
GCTGGTAAAGGTCGAAGCGC	7	0.10
TCGGGTAGGGGCTGTAGCGC	17	0.09
TCTGGGACAGGGCGTAGCGC	20	0.09
TCAGGTAAATGACGTGGCAC	1	0.08

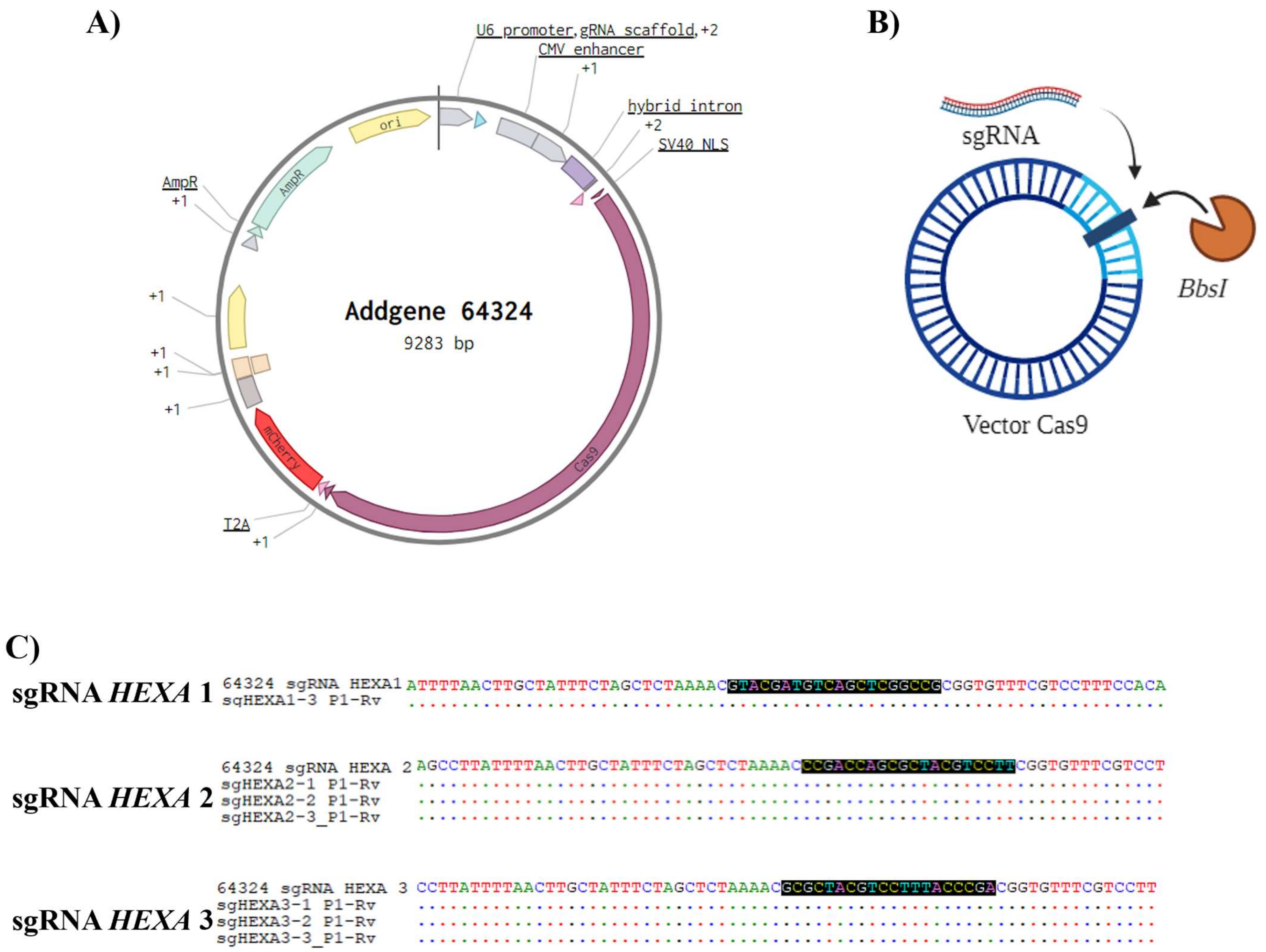

Supplementary Figure S2. **(A)** CRISPR-Cas9 plasmid holding U6 promoter for sgRNA expression, sgRNA scaffold for roper association with the Cas9 enzyme, Cytomegalovirus enhancer (CMV Enhancer), a chimeric intron, a SV40 nuclear localization signal (SV40 NLS), *Streptococcus pyogenes* Cas9 sequence, mCherry reporter sequence, Ampicillin resistance factor (AmpR) and an origin replication site (Ori). Plasmid sequence can be found on the Addgene repository (Addgene # 64324). **(B)** Image summarizing sgRNA cloning into the CRISPR-Cas9 plasmid by enzymatic digestion using a *BbsI* endonuclease for later purification of the linearized plasmid and cloning of sgRNAs by enzymatic ligation. **(C)** Molecular confirmation of sgRNAs sequence by Sanger sequencing. Three sgRNAs were independently cloned into CRISPR-Cas9 plasmid. The predicted sequence of the plasmids holding sgRNA sequence (Top of each alignment) completely align with the sanger sequences. Highlighted letters indicate sgRNA sequences.

Supplementary Table S2. List of primers used in this study for transcriptome validation, evaluation of Endoplasmic reticulum (ER) Stress, assemble, molecular verification of CRISPR-Cas9/sgRNAs plasmids, and T7 mismatch assay

Gene	Sequence (5´-3´)	Function
**TIMP3-Fw**	CGCGTCTATGATGGCAAGATG	Analysis of gene expression for transcriptome validation
**TIMP3-Rv**	ACCGATAGTTCAGCCCCTTG	
**SALL1-Fw**	AGGCACAGGATAGGGGTTCA	
**SALL1-Rv**	CCGGAGAGAGGCCATTCAAG	
**PDPN-Fw**	ATTCGCATCGAGGATCTGCC	
**PDPN-Rv**	CTGTGGCGCTTGGACTTTG	
**CHRDL1-Fw**	TTGCAGGGGTATCGATTGGG	
**CHRDL1-Rv**	GGGCATTTGGCATTGTGGAG	
**GREM1-Fw**	TACGGCCAGTGCAACTCTTT	
**GREM1-Rv**	GGGCAGTTGAGTGTGACCAT	
**CCN1-Fw**	TCATGGTCCCAGTGCTCAAA	
**CCN1-Rv**	TTCTTTCACAAGGCGGCACT	
**CHOP-Fw**	GACTTAAGTCTAAGGCACTG	Gene expression analysis related to Endoplasmic reticulum stress
**CHOP-Rv**	GATACACTTCCTTCTTGAACAC	
**Bip-Fw**	GGTACTGCTTGATGTATGTC	
**Bip-Rv**	GTCTTTCACCTTCATAGACC	
**sgRNA HEXA 1 Fw**	CACCGCGGCCGAGCTGACATCGTAC	Assemble of sgRNAs directed to the *HEXA* gene first exon for later cloning into CRISPR-Cas9 plasmid
**sgRNA HEXA 1 Rv**	AAACGTACGATGTCAGCTCGGCCGC	
**sgRNA HEXA 2 Fw**	CACCGAAGGACGTAGCGCTGGTCGG	
**sgRNA HEXA 2 Rv**	AAACCCGACCAGCGCTACGTCCTTC	
**sgRNA HEXA 3 Fw**	CACCGTCGGGTAAAGGACGTAGCGC	
**sgRNA HEXA 3 Rv**	AAACGCGCTACGTCCTTTACCCGAC	
**Chicken-B-actin AAVS1 sgRNA R´**	CGCTTTTTATAGGGCCGCCGCCGC	PCR confirmation of sgRNA cloning into CRISPR-Cas9 plasmid
**HEXA cut Fw**	TACTTCAGCCTGGCAAGTCCTT	PCR for T7 endonuclease I mismatch assay
**HEXA cut Rv**	GCCCTTGCTCACAGTCTCACTA	

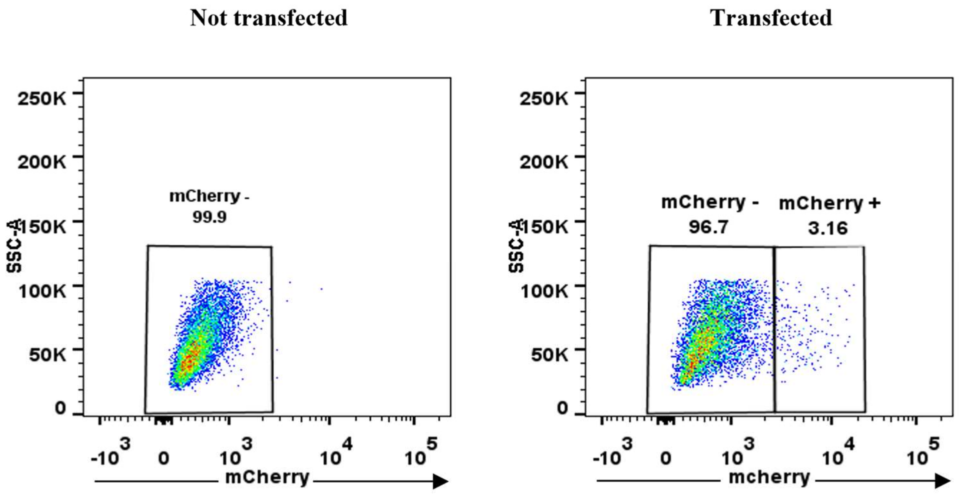

Supplementary Figure S3. U87MG cells were transfected with the CRISPR-Cas9/sgRNA holding the highest cutting efficiency. Transfected cells were sorted by FACS and diluted to a density of 1 cell/well for population expansion and subsequent selection of knocked-out clones.

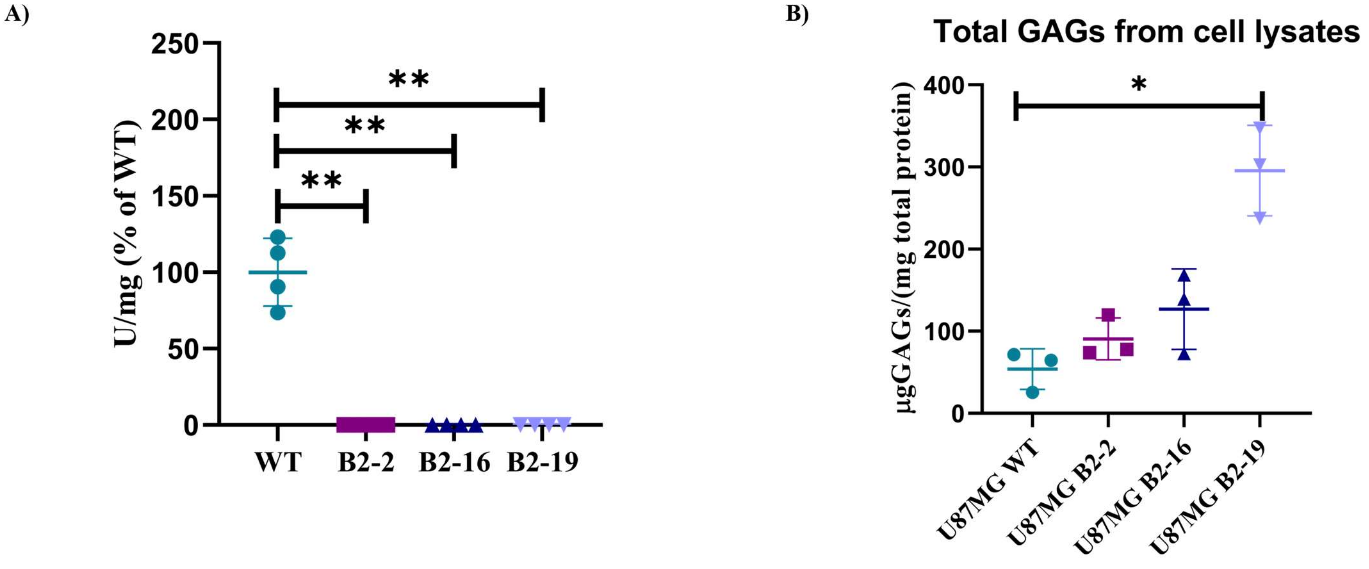

Supplementary Figure S4. Enzymatic HexA measurement and Total GAGs quantification on three U87MG clones after single cell isolation of cells transfected with CRISPR-Cas9/sgRNA *HEXA* plasmid. **(A)** Enzymatic hex-A activity was measured on three B2-2, B2-16 and B2-19 clones isolated from U87MG cell culture transfected with CRISPR-Cas9/sgRNA *HEXA* plasmid. **(B)** Total GAGs were measured from cell lysates of different Hex-A deficient clones. Data are presented as mean ± SEM. Statistical significance was assessed using the Kruskal-Wallis test (** $p < 0.005$) (* $p < 0.05$) followed by Dunn's multiple comparison test.

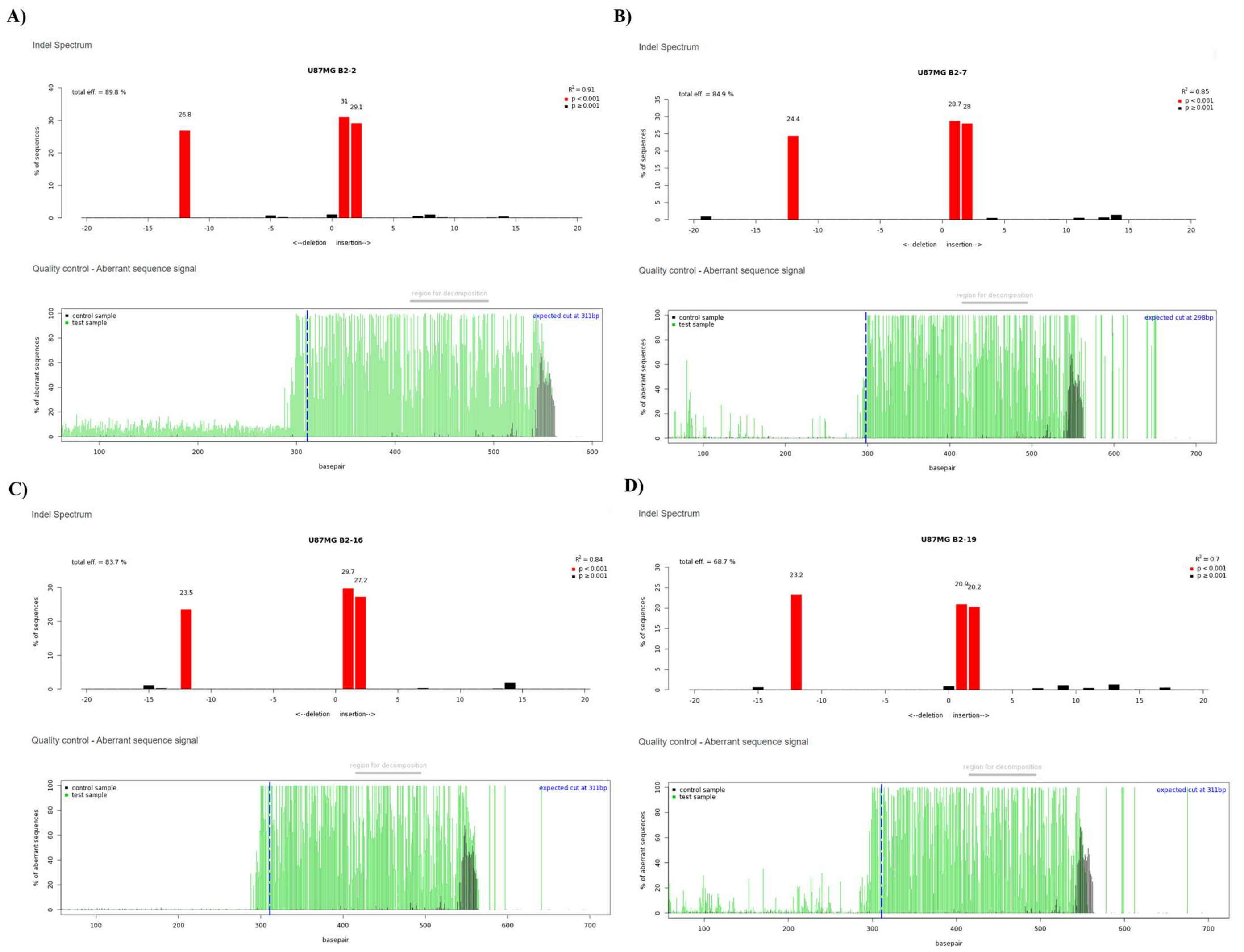

Supplementary Figure S5. Predicted Indel proportion on the *HEXA locus* region sequence obtained from cells transfected with CRISPR-Cas9/sgRNA (Top). Predicted Indels with p<0,001 are shown with red bars. Indel detection analysis by Chromatogram decomposition of sequences from different cell populations (bottom). Chromatogram decomposition of sequences from cells transfected with CRISPR-Cas9 (Green lines) vs untreated control (Black bars). The dotted blue line shows the region where the CRISPR-Cas9 was predicted to cut the genomic *HEXA locus*. The larger the lines the more background noise is expected to be found on the genomic region indicating presence of Indels. Electropherograms of DNA sequences from **(A)** B2-2, **(B)** B2-7, **(C)** B2-16 and **(D)** B2-19 cell cultures were analyzed using the TIDE online tool (https://apps.datacurators.nl/tide/).

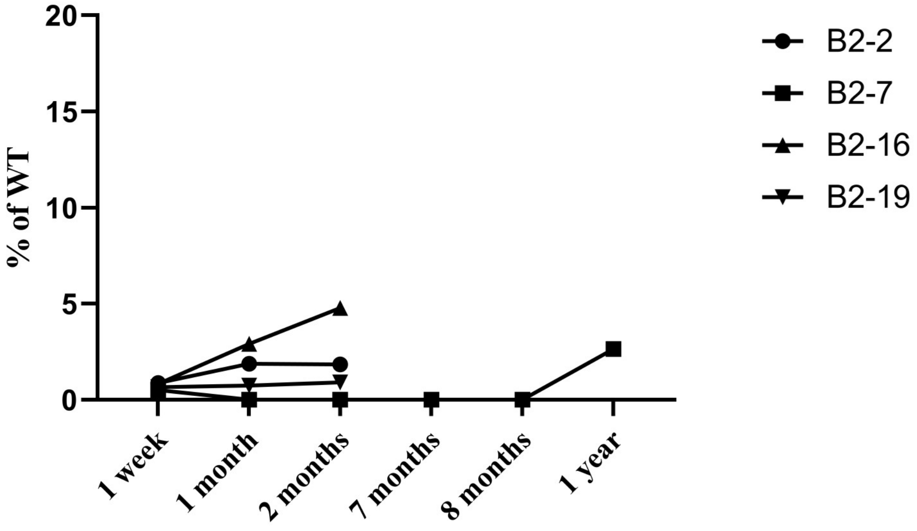

Supplementary Figure S6. HexA enzymatic activity was measured at different time points in lysates from four independent clones with confirmed *HEXA* knockout. Activity is expressed as a percentage of that measured in wild-type (WT) cells (WT = 100%)

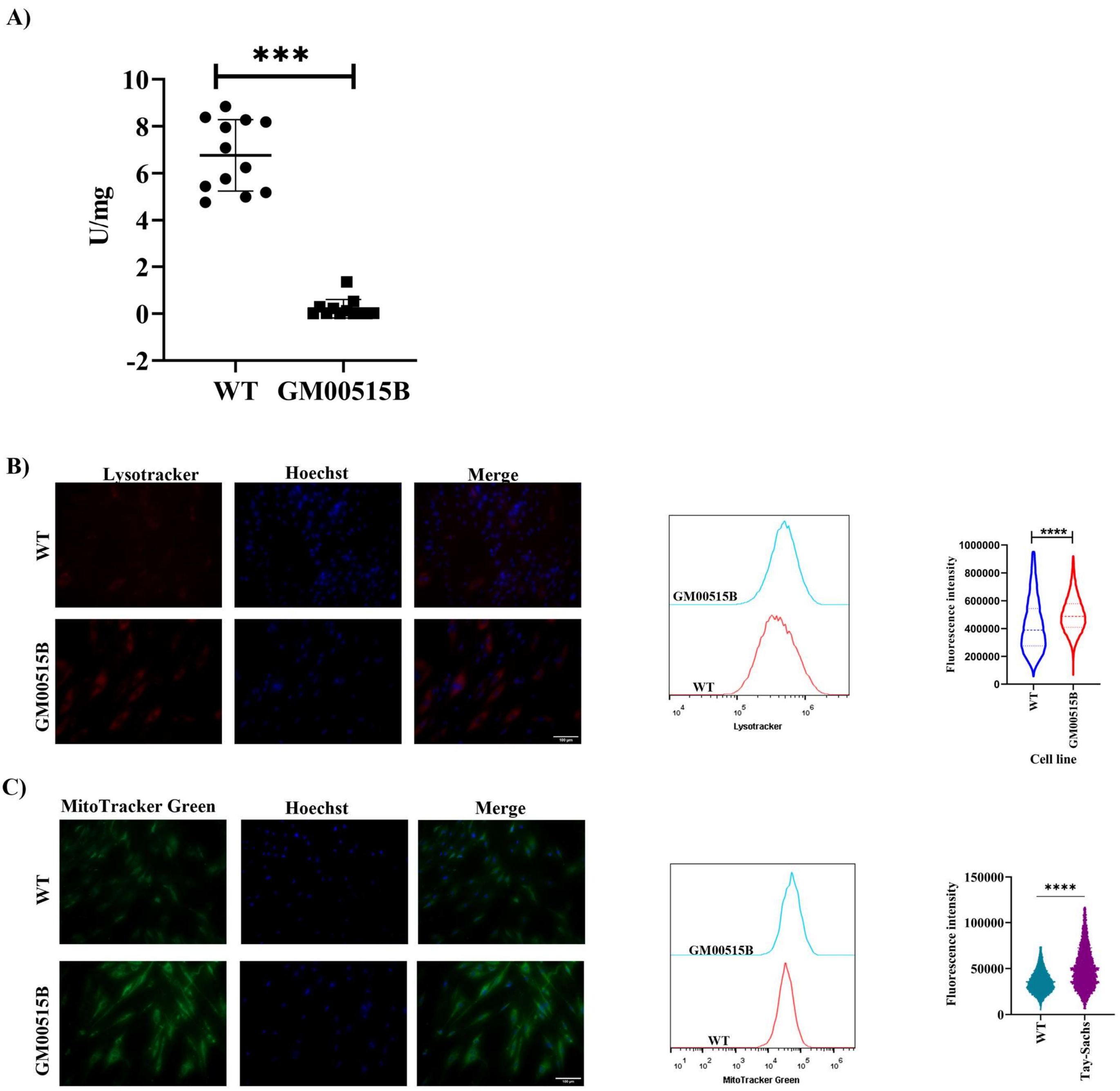

Supplementary Figure S7. (A) Enzymatic hexA activity on unaffected skin fibroblasts and TSD fibroblasts. Enzymatic activity was obtained by 12 independent measurements. **(B)** Lysotracker Deep Red staining for Lysosomal mass visualization on epifluorescence microscopy (Left 20X) and quantification by flow cytometry (Right). **(C)** Cells labeled with MitoTracker green FM for mitochondria staining on epifluorescence microscopy (Left 20X) and quantification by flow cytometry (Right). Data is presented as mean ± SEM. Statistical significance was assessed using the. Mann-Whitney test (*** $p < 0.001$).

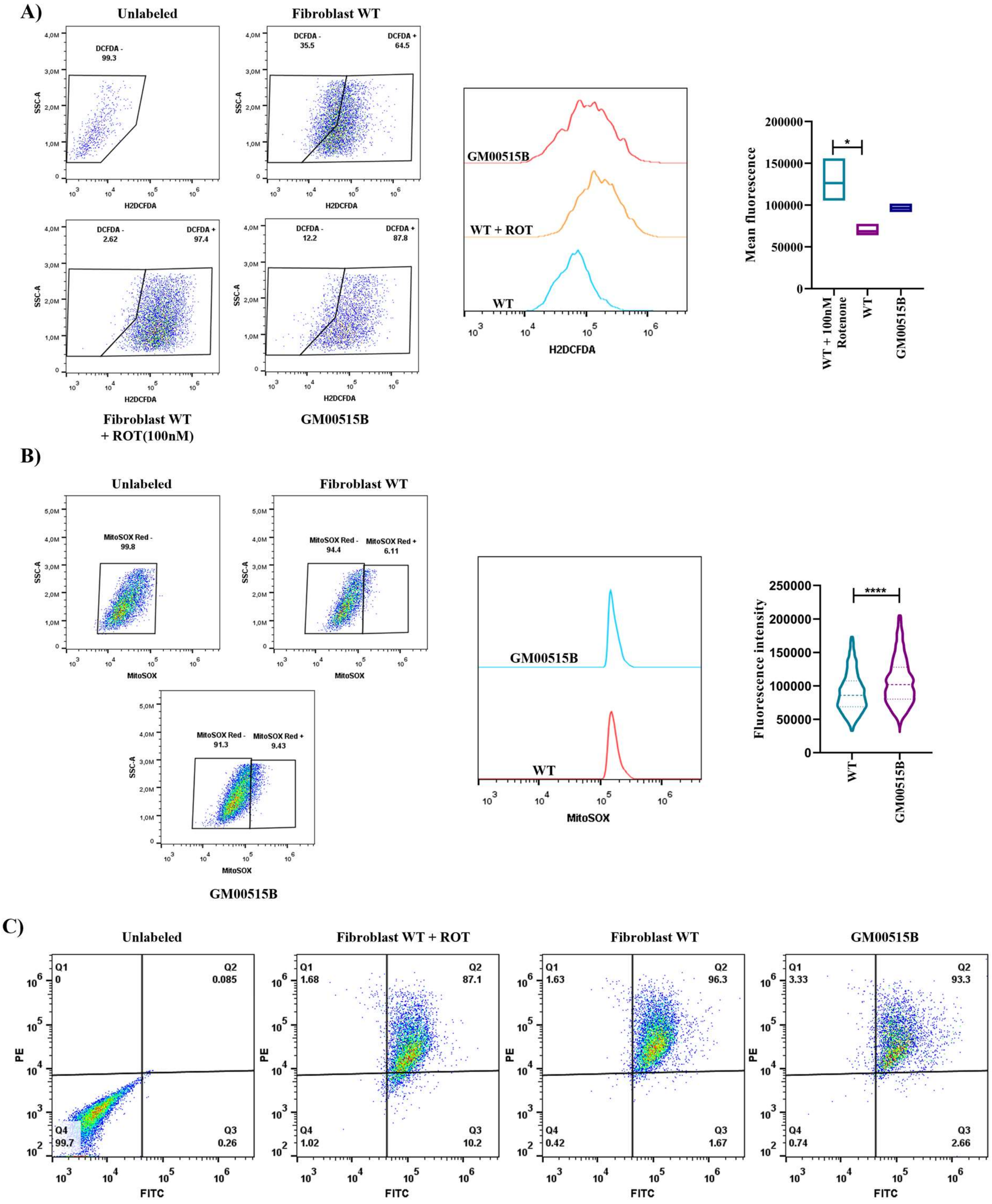

Supplementary Figure S8. Oxidative stress evaluation on TSD skin fibroblasts. **(A)** H_2DCFDA staining for ROS measurement on TSD skin fibroblasts by flow cytometry. Dot plots show increase on fluorescence intensity (Left). Histograms of positive stained cells (middle). Graph showing fluorescence distribution of each cell population (Right) **(B)** MitoSOX fluorescence intensity measurement on TSD skin fibroblasts by flow cytometry. Dot plots show findings on labeled cell with MitoSOX reagent (Left). Histograms of positive stained cells (middle). Graph showing fluorescence distribution of each cell population (Right) **(C)** Representative quadrant plots of non-labeled cells, cell treated with Rotenone (ROT), WT cells, and U87MG B2-7 clone. X axis represents FITC fluorescence and Y axis represent PE fluorescence. Fluorescence quantification by flow cytometry is derived from data of three independent experiments. Data are presented as mean ± SEM. Statistical significance was assessed using the Kruskal–Walli's test (*<0.05) followed by Dunn's multiple comparison test. Mann-Whitney test (**** p < 0.0001).

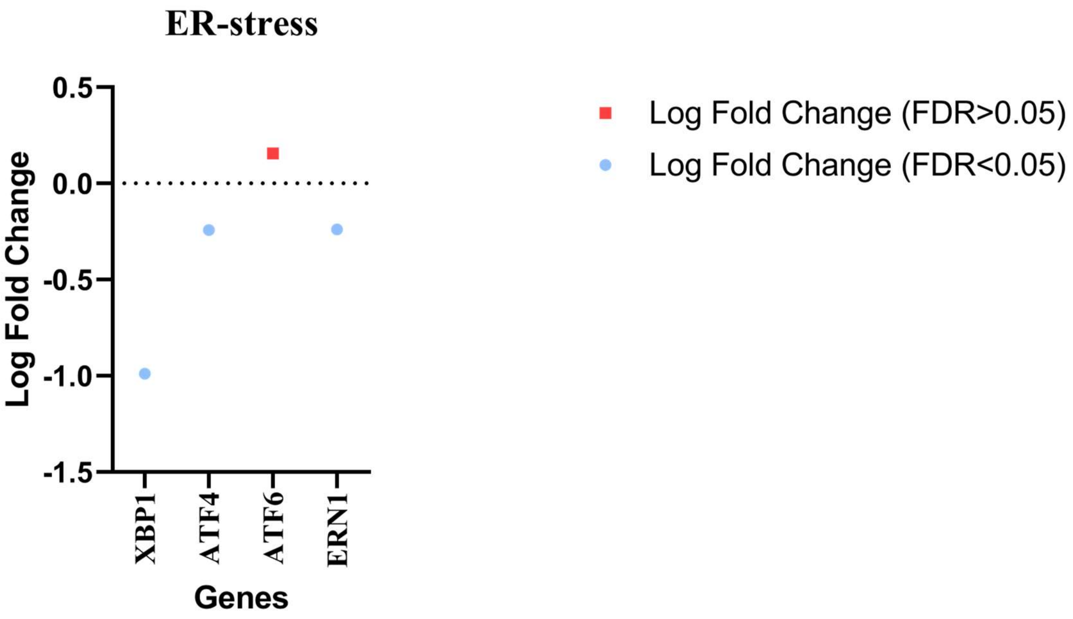

Supplementary Figure S9. Relative gene expression of U87MG B2-7 cells compared to a WT control. Genes shown in the x axis are related to ER-stress. Gene expression is presented as Log_{10} Fold change (compared to control). Statistical significance in gene expression change is considered at FDR <0.05. Fold change and FDR values were obtained from RNA-seq differential expression analysis using three biological replicates per experimental condition.

A)

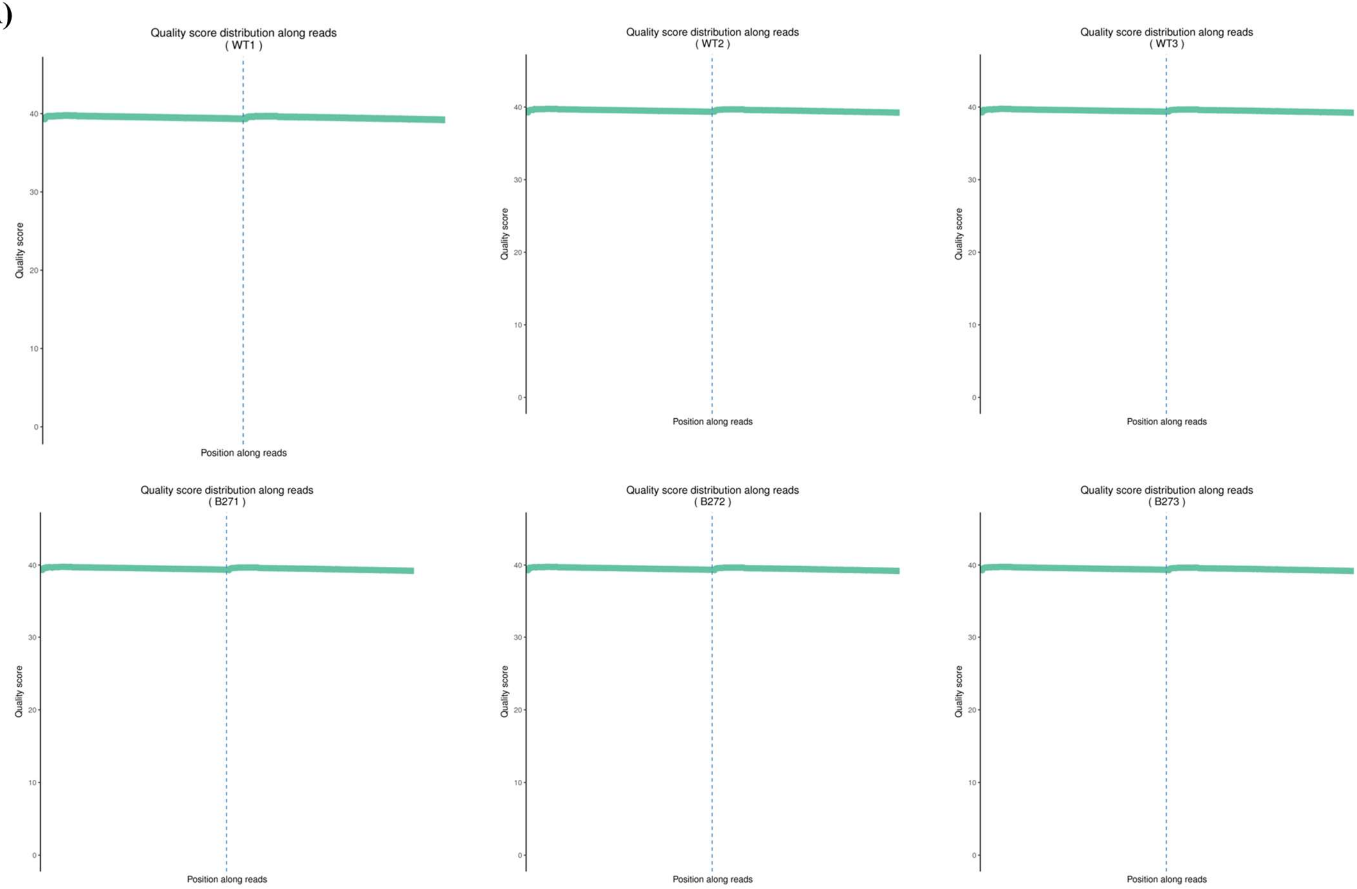

B)

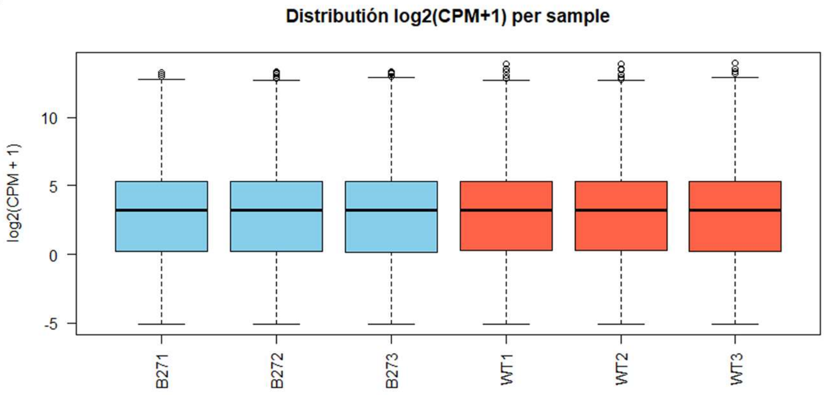

Supplementary Figure S10. (A) Quality scores from RNA samples extracted from U87MG WT and B2-7 clones. **(B)** Quality control boxplot of RNA-Seq counts after normalization by TMM method using edgeR library from R.

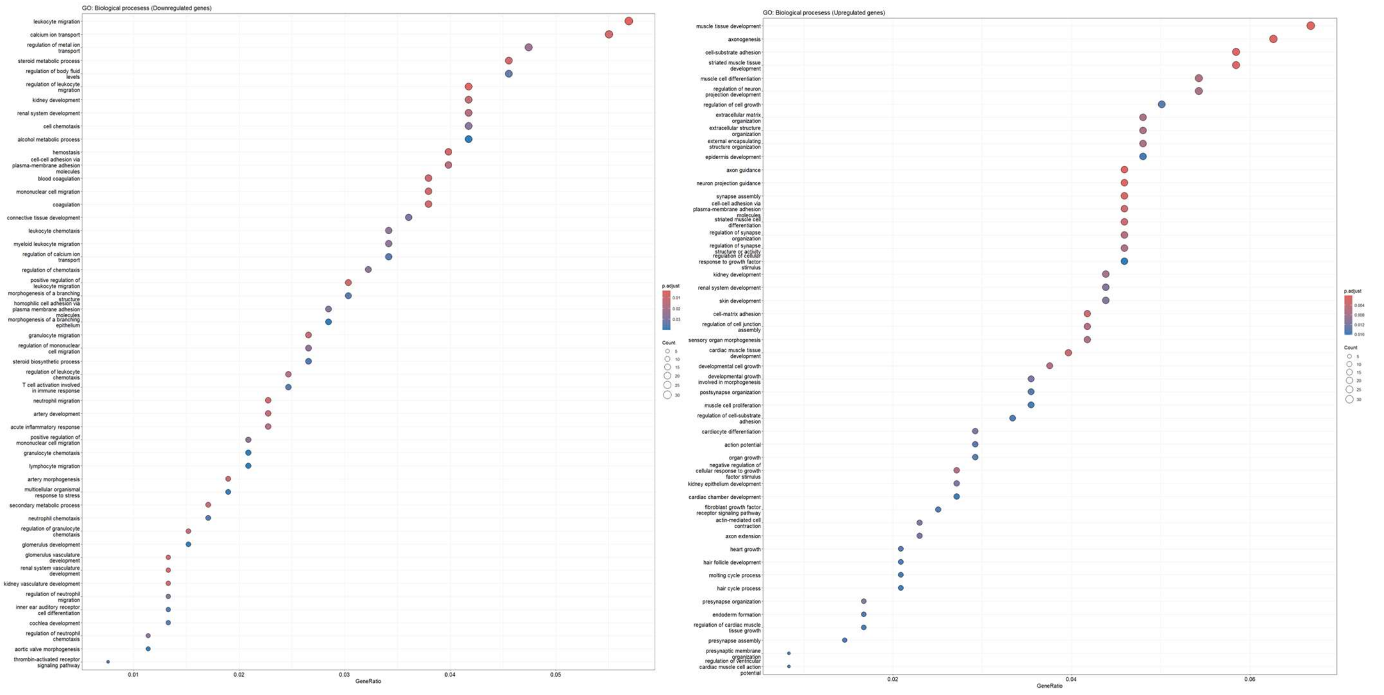

(A) (B)

Supplementary Figure S11. Gene ontology (GO) analysis of the 50 biological processes (BP) terms with genes having most variable expressions on U87MG B2-7 clone compared to the control (WT). **(A)** BP related to downregulated genes are shown on the left and BP related to upregulated genes are on the right **(B).** Gene ratios on x axis are the number of genes on each BP term divided by the total number of genes evaluated. Dots size represents the number of genes involved with each BP term while color represents p-values. P-values<0.05 are considered statistically significant.

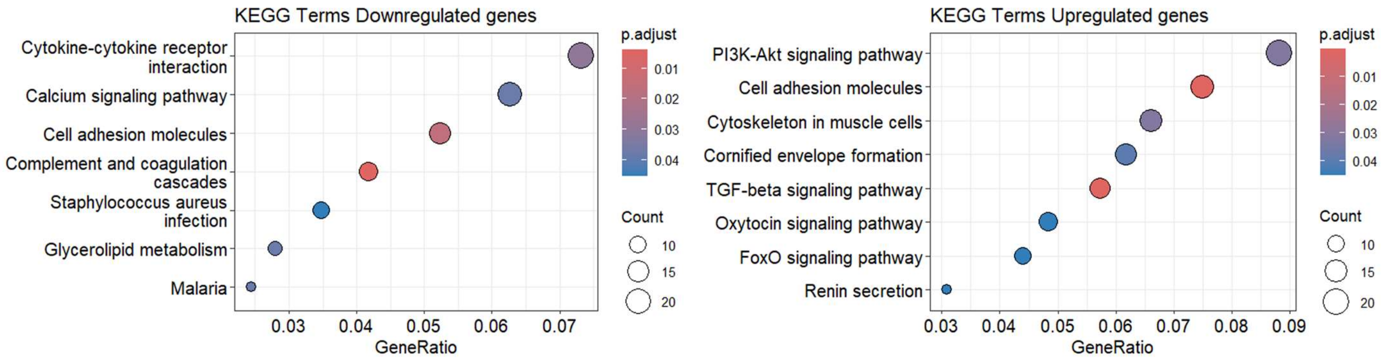

Supplementary Figure S12. Dot plot enrichment analysis based on KEGG terms of downregulated (Left) and upregulated (Right) genes on U87MG B2-7 clone compared to the control Gene ratios on x axis are the number of genes on each KEGG term divided by the total number of genes evaluated. Dots size represents the number of genes involved with each KEGG term while color is representative of p-value significance. P-values<0.05 are considered statistically significant.
